# Supplementary material for: Contrasting cognitive, behavioral, and physiological responses to breathwork vs. naturalistic stimuli in reflective chamber and VR headset environments
Source: PLOS Ment Health. 2025 Mar 12;2(3):e0000269. doi: 10.1371/journal.pmen.0000269 (PMC12798627; doi:10.1371/journal.pmen.0000269)
Supplement: S1 Table — A. MindGym Breathwork. B. MindGym Rain. C. VR Breathwork. D. VR Rain. E. MindGym. F. VR. G. Breathwork. H. Rain. I. All. (PDF) [file pmen.0000269.s001.pdf]

S1: Timepoint effects within groups

Table 1A (MindGym Breathwork)

Group (MindGym Breathwork)													
	Pre			Post			Wilcoxon Signed-Rank test						
DV	n	M	SD	n	M	SD	W	Z	p.raw	p.fdr	RBC	RBC (low)	RBC (high)
TMT (RTACC)	30	135.19	57.76	30	137.86	150.46	337.00	2.15	0.031	0.0543	0.45	0.07	0.71
Architex (Total Speed)	30	193.91	87.87	30	142.14	60.94	396.00	3.36	< .001	0.0007	0.70	0.43	0.86
Architex (Total Score)	30	39.07	3.22	30	38.13	3.34	258.50	1.67	0.097	0.1132	0.37	-0.05	0.67
Anxiety (STAI)	31	35.45	8.63	31	31.77	8.86	323.50	2.29	0.022	0.0513	0.49	0.12	0.74
Arousal	31	2.61	1.52	31	2.42	1.46	130.50	0.52	0.609	0.6090	0.13	-0.34	0.55
Valence	31	6.71	1.87	31	7.74	2.03	57.00	-2.03	0.042	0.0588	-0.51	-0.78	-0.07
POMS	31	12.45	24.28	31	2.55	22.53	331.00	2.92	0.004	0.0140	0.63	0.31	0.82

Table 1A. Nonparametric t-test (Wilcoxon Signed-Rank) of timepoint (pre vs post) differences for the MindGym (Breathwork) group. 'DV' refers to dependent variable. 'n' refers to group sample size. 'M' and 'SD' refer to mean and standard deviation. 'p.raw' and 'p.fdr' refer to the uncorrected p value and FDR-corrected p value. 'RBC' refers to rank biserial correlation as a measure of effect size (ranging from -1 to 1) and reflects the relative proportions of positive and negative ranks. 'RBC (low)' and 'RBC (high)' refer to the lower and upper bounds of the 95% confidence interval of RBC.

Table 1B (MindGym Rain)

Group (MindGym Rain)													
	Pre			Post			Wilcoxon Signed-Rank test						
DV	n	M	SD	n	M	SD	W	Z	p.raw	p.fdr	RBC	RBC (low)	RBC (high)
TMT (RTACC)	31	169.98	113.92	31	127.34	53.43	411.00	3.19	< .001	0.0002	0.66	0.37	0.83
Architex (Total Speed)	30	189.86	78.98	30	116.97	42.04	421.00	4.40	< .001	0.0002	0.94	0.86	0.97
Architex (Total Score)	30	37.40	7.53	30	38.05	7.93	99.50	-1.44	0.152	0.1773	-0.34	-0.67	0.11
Anxiety (STAI)	32	37.59	9.01	32	33.50	6.08	353.50	2.94	0.003	0.0053	0.63	0.31	0.82
Arousal	32	3.06	2.30	32	2.69	1.75	140.50	0.46	0.657	0.6570	0.11	-0.35	0.53
Valence	32	7.44	1.50	32	7.84	1.94	75.50	-1.90	0.054	0.0756	-0.45	-0.74	-0.02
POMS	32	15.47	22.05	32	4.97	16.69	442.00	3.80	< .001	0.0002	0.78	0.57	0.90

Table 1B. Nonparametric t-test (Wilcoxon Signed-Rank) of timepoint (pre vs post) differences for the MindGym (Rain) group. 'DV' refers to dependent variable. 'n' refers to group sample size. 'M' and 'SD' refer to mean and standard deviation. 'p.raw' and 'p.fdr' refer to the uncorrected p value and FDR-corrected p value. 'RBC' refers to rank biserial correlation as a measure of effect size (ranging from -1 to 1) and reflects the relative proportions of positive and negative ranks. 'RBC (low)' and 'RBC (high)' refer to the lower and upper bounds of the 95% confidence interval of RBC.

Table 1C (VR Breathwork)

Group (VR Breathwork)													
	Pre			Post			Wilcoxon Signed-Rank test						
DV	n	M	SD	n	M	SD	W	Z	p.raw	p.fdr	RBC	RBC (low)	RBC (high)
TMT (RTACC)	33	195.94	200.00	31	152.73	96.38	329.00	1.59	0.116	0.2030	0.33	-0.06	0.63

Architex (Total Speed)	27	246.99	300.40	27	132.03	37.98	355.00	3.99	< .001	0.0004	0.88	0.73	0.95
Architex (Total Score)	27	38.30	3.31	27	37.93	3.65	188.00	1.09	0.282	0.3290	0.25	-0.20	0.62
Anxiety (STAI)	33	36.64	11.01	33	30.94	7.31	405.00	3.55	< .001	0.0004	0.74	0.50	0.88
Arousal	33	2.55	1.82	33	3.24	2.11	135.00	-1.30	0.195	0.2730	-0.29	-0.62	0.14
Valence	33	7.46	1.39	33	7.33	2.80	176.50	-0.30	0.772	0.7720	-0.07	-0.46	0.35
POMS	33	12.79	29.37	33	2.21	23.77	406.00	3.10	0.002	0.0047	0.64	0.34	0.82

Table 1C. Nonparametric t-test (Wilcoxon Signed-Rank) of timepoint (pre vs post) differences for the VR (Breathwork) group. ‘DV’ refers to dependent variable. ‘n’ refers to group sample size. ‘M’ and ‘SD’ refer to mean and standard deviation. ‘p.raw’ and ‘p.fdr’ refer to the uncorrected p value and FDR-corrected p value. ‘RBC’ refers to rank biserial correlation as a measure of effect size (ranging from -1 to 1) and reflects the relative proportions of positive and negative ranks. ‘RBC (low)’ and ‘RBC (high)’ refer to the lower and upper bounds of the 95% confidence interval of RBC.

Table 1D (VR Rain)

Group (VR Rain)													
	Pre			Post			Wilcoxon Signed-Rank test						
**DV**	**n**	**M**	**SD**	**n**	**M**	**SD**	**W**	**Z**	**p.raw**	**p.fdr**	**RBC**	**RBC (low)**	**RBC (high)**
TMT (RTACC)	30	161.35	203.67	30	125.93	91.44	308.00	1.55	0.124	0.3523	0.33	-0.07	0.63
Architex (Total Speed)	28	217.82	89.32	28	148.57	67.71	379.00	4.01	< .001	0.0007	0.87	0.72	0.94
Architex (Total Score)	28	38.34	2.58	28	37.38	3.47	197.50	0.94	0.352	0.4928	0.22	-0.23	0.58
Anxiety (STAI)	30	34.83	9.88	30	33.40	6.77	235.00	0.73	0.473	0.5518	0.16	-0.26	0.53
Arousal	30	3.50	2.66	30	2.87	2.16	208.50	1.24	0.215	0.3763	0.28	-0.16	0.63
Valence	30	7.60	1.83	30	7.63	1.73	122.50	-0.13	0.908	0.9080	-0.03	-0.47	0.42
POMS	30	13.17	31.32	30	6.00	23.07	266.50	1.45	0.151	0.3523	0.31	-0.10	0.63

Table 1D. Nonparametric t-test (Wilcoxon Signed-Rank) of timepoint (pre vs post) differences for the VR (Rain) group. ‘DV’ refers to dependent variable. ‘n’ refers to group sample size. ‘M’ and ‘SD’ refer to mean and standard deviation. ‘p.raw’ and ‘p.fdr’ refer to the uncorrected p value and FDR-corrected p value. ‘RBC’ refers to rank biserial correlation as a measure of effect size (ranging from -1 to 1) and reflects the relative proportions of positive and negative ranks. ‘RBC (low)’ and ‘RBC (high)’ refer to the lower and upper bounds of the 95% confidence interval of RBC.

Table 1E (MindGym)

Group (MindGym)													
	Pre			Post			Wilcoxon Signed-Rank test						
**DV**	**n**	**M**	**SD**	**n**	**M**	**SD**	**W**	**Z**	**p.raw**	**p.fdr**	**RBC**	**RBC (low)**	**RBC (high)**
TMT (RTACC)	61	152.87	91.70	61	132.51	111.34	1470.00	3.77	< .001	0.0002	0.56	0.32	0.72
Architex (Total Speed)	60	191.88	82.86	60	129.55	53.43	1613.00	5.50	< .001	0.0002	0.82	0.70	0.90
Architex (Total Score)	60	38.23	5.80	60	38.09	6.03	702.50	0.37	0.714	0.7140	0.06	-0.25	0.36
Anxiety (STAI)	63	36.54	8.82	63	32.65	7.57	1328.00	3.66	< .001	0.0002	0.55	0.32	0.73
Arousal	63	2.84	1.95	63	2.56	1.60	529.50	0.68	0.493	0.5752	0.12	-0.22	0.43
Valence	63	7.08	1.72	63	7.79	1.97	260.00	-2.74	0.006	0.0084	-0.48	-0.69	-0.18
POMS	63	13.98	23.03	63	3.78	19.66	1516.50	4.77	< .001	0.0002	0.71	0.54	0.83

Table 1E. Nonparametric t-test (Wilcoxon Signed-Rank) of timepoint (pre vs post) differences for the MindGym group. ‘DV’ refers to dependent variable. ‘n’ refers to group sample size. ‘M’ and ‘SD’ refer to mean and standard deviation. ‘p.raw’ and ‘p.fdr’ refer to the uncorrected p value and FDR-corrected p value. ‘RBC’ refers to rank biserial correlation as a measure of effect size (ranging from -1

to 1) and reflects the relative proportions of positive and negative ranks. 'RBC (low)' and 'RBC (high)' refer to the lower and upper bounds of the 95% confidence interval of RBC.

Table 1F (VR)

Group (VR)													
	Pre			Post			Wilcoxon Signed-Rank test						
DV	n	M	SD	n	M	SD	W	Z	p.raw	p.fdr	RBC	RBC (low)	RBC (high)
TMT (RTACC)	63	179.47	200.88	61	139.55	94.17	1253.00	2.21	0.027	0.0473	0.33	0.05	0.56
Architex (Total Speed)	55	232.14	218.30	55	140.45	55.28	1439.00	5.61	< .001	0.0004	0.87	0.77	0.93
Architex (Total Score)	55	38.32	2.93	55	37.65	3.54	754.50	1.41	0.158	0.2212	0.23	-0.09	0.51
Anxiety (STAI)	63	35.78	10.44	63	32.11	7.11	1261.00	3.14	0.002	0.0047	0.47	0.22	0.67
Arousal	63	3.00	2.29	63	3.06	2.12	694.00	0.05	0.967	0.9670	0.01	-0.30	0.31
Valence	63	7.52	1.61	63	7.48	2.34	574.00	-0.38	0.703	0.8202	-0.06	-0.37	0.25
POMS	63	12.97	30.06	63	4.02	23.33	1326.50	3.33	< .001	0.0004	0.50	0.25	0.69

Table 1F. Nonparametric t-test (Wilcoxon Signed-Rank) of timepoint (pre vs post) differences for the VR group. 'DV' refers to dependent variable. 'n' refers to group sample size. 'M' and 'SD' refer to mean and standard deviation. 'p.raw' and 'p.fdr' refer to the uncorrected p value and FDR-corrected p value. 'RBC' refers to rank biserial correlation as a measure of effect size (ranging from -1 to 1) and reflects the relative proportions of positive and negative ranks. 'RBC (low)' and 'RBC (high)' refer to the lower and upper bounds of the 95% confidence interval of RBC.

Table 1G (Breathwork)

Group (Breathwork)													
	Pre			Post			Wilcoxon Signed-Rank test						
DV	n	M	SD	n	M	SD	W	Z	p.raw	p.fdr	RBC	RBC (low)	RBC (high)
TMT (RTACC)	63	167.01	152.12	61	145.42	125.07	1315.00	2.65	0.008	0.0140	0.39	0.12	0.61
Architex (Total Speed)	57	219.05	215.90	57	137.35	51.17	1480.00	5.19	< .001	0.0002	0.79	0.65	0.88
Architex (Total Score)	57	38.70	3.25	57	38.04	3.46	870.00	1.94	0.052	0.0728	0.31	0.01	0.56
Anxiety (STAI)	64	36.06	9.87	64	31.34	8.05	1435.00	4.15	< .001	0.0002	0.62	0.41	0.77
Arousal	64	2.58	1.67	64	2.84	1.85	519.00	-0.71	0.478	0.4780	-0.12	-0.42	0.20
Valence	64	7.09	1.67	64	7.53	2.45	436.00	-1.56	0.118	0.1377	-0.26	-0.53	0.06
POMS	64	12.63	26.81	64	2.38	23.00	1448.00	4.25	< .001	0.0002	0.64	0.43	0.78

Table 1G. Nonparametric t-test (Wilcoxon Signed-Rank) of timepoint (pre vs post) differences for the Breathwork group. 'DV' refers to dependent variable. 'n' refers to group sample size. 'M' and 'SD' refer to mean and standard deviation. 'p.raw' and 'p.fdr' refer to the uncorrected p value and FDR-corrected p value. 'RBC' refers to rank biserial correlation as a measure of effect size (ranging from -1 to 1) and reflects the relative proportions of positive and negative ranks. 'RBC (low)' and 'RBC (high)' refer to the lower and upper bounds of the 95% confidence interval of RBC.

Table 1H (Rain)

Group (Rain)													
	Pre			Post			Wilcoxon Signed-Rank test						
DV	n	M	SD	n	M	SD	W	Z	p.raw	p.fdr	RBC	RBC (low)	RBC (high)
TMT (RTACC)	61	165.74	162.97	61	126.65	73.95	1429.00	3.47	< .001	0.0002	0.51	0.27	0.69

Architex (Total Speed)	58	203.36	84.57	58	132.23	57.66	1567.00	5.88	< .001	0.0002	0.90	0.82	0.94
Architex (Total Score)	58	37.85	5.68	58	37.72	6.15	586.50	-0.26	0.799	0.7990	-0.04	-0.35	0.27
Anxiety (STAI)	62	36.26	9.46	62	33.45	6.37	1156.50	2.62	0.009	0.0158	0.40	0.12	0.62
Arousal	62	3.27	2.47	62	2.77	1.95	686.50	1.30	0.192	0.2240	0.22	-0.11	0.50
Valence	62	7.52	1.66	62	7.74	1.83	395.50	-1.38	0.162	0.2240	-0.24	-0.52	0.10
POMS	62	14.36	26.73	62	5.47	19.87	1403.50	3.91	< .001	0.0002	0.59	0.36	0.75

Table 1H. Nonparametric t-test (Wilcoxon Signed-Rank) of timepoint (pre vs post) differences for the Rain group. 'DV' refers to dependent variable. 'n' refers to group sample size. 'M' and 'SD' refer to mean and standard deviation. 'p.raw' and 'p.fdr' refer to the uncorrected p value and FDR-corrected p value. 'RBC' refers to rank biserial correlation as a measure of effect size (ranging from -1 to 1) and reflects the relative proportions of positive and negative ranks. 'RBC (low)' and 'RBC (high)' refer to the lower and upper bounds of the 95% confidence interval of RBC.

Table 1I (All)

**Group (All)**													
	**Pre**			**Post**			**Wilcoxon Signed-Rank test**						
**DV**	**n**	**M**	**SD**	**n**	**M**	**SD**	**W**	**Z**	**p.raw**	**p.fdr**	**RBC**	**RBC (low)**	**RBC (high)**
TMT (RTACC)	124	166.38	156.91	122	136.03	102.75	5443.00	4.32	< .001	0.0002	0.45	0.27	0.60
Architex (Total Speed)	115	211.14	162.89	115	134.76	54.36	6044.00	7.82	< .001	0.0002	0.84	0.77	0.90
Architex (Total Score)	115	38.27	4.64	115	37.88	4.98	2886.00	1.24	0.214	0.2497	0.14	-0.08	0.35
Anxiety (STAI)	126	36.16	9.63	126	32.38	7.32	5156.50	4.86	< .001	0.0002	0.52	0.35	0.66
Arousal	126	2.92	2.12	126	2.81	1.89	2418.00	0.51	0.606	0.6060	0.06	-0.17	0.28
Valence	126	7.30	1.67	126	7.64	2.16	1626.00	-2.14	0.030	0.0420	-0.26	-0.46	-0.03
POMS	126	13.48	26.68	126	3.90	21.49	5659.00	5.77	< .001	0.0002	0.61	0.47	0.73

Table 1I. Nonparametric t-test (Wilcoxon Signed-Rank) of timepoint (pre vs post) differences for all groups combined. 'DV' refers to dependent variable. 'n' refers to group sample size. 'M' and 'SD' refer to mean and standard deviation. 'p.raw' and 'p.fdr' refer to the uncorrected p value and FDR-corrected p value. 'RBC' refers to rank biserial correlation as a measure of effect size (ranging from -1 to 1) and reflects the relative proportions of positive and negative ranks. 'RBC (low)' and 'RBC (high)' refer to the lower and upper bounds of the 95% confidence interval of RBC.
