# Supplementary material for: Contrasting cognitive, behavioral, and physiological responses to breathwork vs. naturalistic stimuli in reflective chamber and VR headset environments
Source: PLOS Ment Health. 2025 Mar 12;2(3):e0000269. doi: 10.1371/journal.pmen.0000269 (PMC12798627; doi:10.1371/journal.pmen.0000269)
Supplement: S2 Table — A. MindGym Breathwork vs MindGym Rain. B. MindGym Breathwork vs VR Breathwork. C. MindGym Rain vs VR Rain. D. VR Breathwork vs VR Rain. E. Breathwork vs Rain. F. MindGym vs VR. (PDF) [file pmen.0000269.s002.pdf]

S2: Group differences in timepoint effects

Table 2A (MindGym Breathwork vs MindGym Rain)

	MindGym (Breathwork)			MindGym (Rain)			Mann-Whitney U test					
DV	n	ΔM	SD	n	ΔM	SD	W	p.raw	p.fdr	RBC	RBC (low)	RBC (high)
TMT (RTACC)	30	2.68	157.82	31	-42.64	95.63	514	0.487	0.713	0.11	-0.18	0.38
Architex (Total Speed)	30	-51.77	67.90	30	-72.88	61.68	512	0.366	0.713	0.14	-0.16	0.41
Architex (Total Score)	30	-0.93	2.97	30	0.65	2.50	298.5	0.025	0.175	-0.34	-0.57	-0.06
Anxiety (STAI)	31	-3.68	9.35	32	-4.09	8.65	475.5	0.783	0.783	-0.04	-0.32	0.24
Arousal	31	-0.19	1.83	32	-0.38	2.30	471.5	0.736	0.783	-0.05	-0.33	0.23
Valence	31	1.03	2.76	32	0.41	2.03	546	0.486	0.713	0.10	-0.18	0.37
POMS	31	-9.90	17.48	32	-10.50	13.34	544.5	0.509	0.713	0.10	-0.19	0.37

Table 2A. Nonparametric t-tests (Mann-Whitney U) between the MindGym (Breathwork) and MindGym (Rain) groups. 'DV' refers to dependent variable. 'n' refers to group sample size. 'ΔM' and 'SD' refer to mean and standard deviation of the difference score (post – pre). 'p.raw' and 'p.fdr' refer to the uncorrected p value and FDR-corrected p value. 'RBC' refers to rank biserial correlation as a measure of effect size (ranging from -1 to 1) and reflects the relative proportions of positive and negative ranks. 'RBC (low)' and 'RBC (high)' refer to the lower and upper bounds of the 95% confidence interval of RBC.

Table 2B (MindGym Breathwork vs VR Breathwork)

	MindGym (Breathwork)			VR (Breathwork)			Mann-Whitney U test					
DV	n	ΔM	SD	n	ΔM	SD	W	p.raw	p.fdr	RBC	RBC (low)	RBC (high)
TMT (RTACC)	30	2.68	157.82	31	-35.06	176.44	416.00	0.487	0.852	-0.11	-0.38	0.18
Architex (Total Speed)	30	-51.77	67.90	27	-114.97	301.49	433.00	0.663	0.856	0.07	-0.23	0.36
Architex (Total Score)	30	-0.93	2.97	27	-0.37	2.55	357.00	0.446	0.852	-0.12	-0.40	0.18
Anxiety (STAI)	31	-3.68	9.35	33	-5.70	8.41	534.00	0.767	0.856	0.04	-0.24	0.32
Arousal	31	-0.19	1.83	33	0.70	2.76	417.50	0.203	0.852	-0.18	-0.44	0.10
Valence	31	1.03	2.76	33	-0.12	3.23	582.00	0.341	0.852	0.14	-0.15	0.40
POMS	31	-9.90	17.48	33	-10.58	20.44	525.50	0.856	0.856	0.03	-0.25	0.30

Table 2B. Nonparametric t-tests (Mann-Whitney U) between the MindGym (Breathwork) and VR (Breathwork) groups. 'DV' refers to dependent variable. 'n' refers to group sample size. 'ΔM' and 'SD' refer to mean and standard deviation of the difference score (post – pre). 'p.raw' and 'p.fdr' refer to the uncorrected p value and FDR-corrected p value. 'RBC' refers to rank biserial correlation as a measure of effect size (ranging from -1 to 1) and reflects the relative proportions of positive and negative ranks. 'RBC (low)' and 'RBC (high)' refer to the lower and upper bounds of the 95% confidence interval of RBC.

Table 2C (MindGym Rain vs VR Rain)

	MindGym (Rain)			VR (Rain)			Mann-Whitney U test					
DV	n	ΔM	SD	n	ΔM	SD	W	p.raw	p.fdr	RBC	RBC (low)	RBC (high)
TMT (RTACC)	31	-42.64	95.63	30	-35.42	206.73	347.00	0.090	0.214	-0.25	-0.50	0.03
Architex (Total Speed)	30	-72.88	61.68	28	-69.26	73.95	409.00	0.871	0.871	-0.03	-0.32	0.27
Architex (Total Score)	30	0.65	2.50	28	-0.96	3.74	519.50	0.122	0.214	0.24	-0.06	0.49
Anxiety (STAI)	32	-4.09	8.65	30	-1.43	7.17	367.50	0.114	0.214	-0.23	-0.48	0.05

Arousal	32	-0.38	2.30	30	-0.63	2.87	521.50	0.557	0.650	0.09	-0.20	0.36
Valence	32	0.41	2.03	30	0.03	1.81	559.50	0.256	0.358	0.17	-0.12	0.43
POMS	32	-10.50	13.34	30	-7.17	19.80	350.50	0.069	0.214	-0.27	-0.51	0.01

Table 2C. Nonparametric t-tests (Mann-Whitney U) between the MindGym (Rain) and VR (Rain) groups. 'DV' refers to dependent variable. 'n' refers to group sample size. 'ΔM' and 'SD' refer to mean and standard deviation of the difference score (post – pre). 'p.raw' and 'p.fdr' refer to the uncorrected p value and FDR-corrected p value. 'RBC' refers to rank biserial correlation as a measure of effect size (ranging from -1 to 1) and reflects the relative proportions of positive and negative ranks. 'RBC (low)' and 'RBC (high)' refer to the lower and upper bounds of the 95% confidence interval of RBC.

Table 2D (VR Breathwork vs VR Rain)

	VR (Breathwork)			VR (Rain)			Mann-Whitney U test					
**DV**	**n**	**ΔM**	**SD**	**n**	**ΔM**	**SD**	**W**	**p.raw**	**p.fdr**	**RBC**	**RBC (low)**	**RBC (high)**
TMT (RTACC)	31	-35.06	176.44	30	-35.42	206.73	456.00	0.903	0.939	-0.02	-0.30	0.27
Architex (Total Speed)	27	-114.97	301.49	28	-69.26	73.95	395.00	0.783	0.939	0.05	-0.26	0.34
Architex (Total Score)	27	-0.37	2.55	28	-0.96	3.74	373.00	0.939	0.939	-0.01	-0.31	0.29
Anxiety (STAI)	33	-5.70	8.41	30	-1.43	7.17	347.00	0.042	0.294	-0.30	-0.53	-0.02
Arousal	33	0.70	2.76	30	-0.63	2.87	610.50	0.110	0.385	0.23	-0.05	0.48
Valence	33	-0.12	3.23	30	0.03	1.81	533.00	0.601	0.939	0.08	-0.21	0.35
POMS	33	-10.58	20.44	30	-7.17	19.80	408.50	0.236	0.551	-0.18	-0.43	0.11

Table 2D. Nonparametric t-tests (Mann-Whitney U) between the VR (Breathwork) and VR (Rain) groups. 'DV' refers to dependent variable. 'n' refers to group sample size. 'ΔM' and 'SD' refer to mean and standard deviation of the difference score (post – pre). 'p.raw' and 'p.fdr' refer to the uncorrected p value and FDR-corrected p value. 'RBC' refers to rank biserial correlation as a measure of effect size (ranging from -1 to 1) and reflects the relative proportions of positive and negative ranks. 'RBC (low)' and 'RBC (high)' refer to the lower and upper bounds of the 95% confidence interval of RBC.

Table 2E (Breathwork vs Rain)

	Breathwork			Rain			Mann-Whitney U test					
**DV**	**n**	**ΔM**	**SD**	**n**	**ΔM**	**SD**	**W**	**p.raw**	**p.fdr**	**RBC**	**RBC (low)**	**RBC (high)**
TMT (RTACC)	61	-16.50	167.23	61	-39.09	158.87	1936.00	0.701	0.705	0.04	-0.16	0.24
Architex (Total Speed)	57	-81.71	213.55	58	-71.13	67.30	1804.00	0.400	0.560	0.09	-0.12	0.30
Architex (Total Score)	57	-0.67	2.77	58	-0.13	3.24	1353.50	0.094	0.480	-0.18	-0.38	0.03
Anxiety (STAI)	64	-4.72	8.86	62	-2.81	8.02	1679.00	0.137	0.480	-0.15	-0.34	0.05
Arousal	64	0.27	2.38	62	-0.50	2.57	2192.50	0.304	0.560	0.11	-0.10	0.30
Valence	64	0.44	3.04	62	0.23	1.92	2162.00	0.379	0.560	0.09	-0.11	0.28
POMS	64	-10.25	18.92	62	-8.89	16.73	1906.00	0.705	0.705	-0.04	-0.24	0.16

Table 2E. Nonparametric t-tests (Mann-Whitney U) between the Breathwork and Rain groups. 'DV' refers to dependent variable. 'n' refers to group sample size. 'ΔM' and 'SD' refer to mean and standard deviation of the difference score (post – pre). 'p.raw' and 'p.fdr' refer to the uncorrected p value and FDR-corrected p value. 'RBC' refers to rank biserial correlation as a measure of effect size (ranging from -1 to 1) and reflects the relative proportions of positive and negative ranks. 'RBC (low)' and 'RBC (high)' refer to the lower and upper bounds of the 95% confidence interval of RBC.

Table 2F (MindGym vs VR)

	MindGym			VR			Mann-Whitney U test					
**DV**	**n**	**ΔM**	**SD**	**n**	**ΔM**	**SD**	**W**	**p.raw**	**p.fdr**	**RBC**	**RBC (low)**	**RBC (high)**
TMT (RTACC)	61	-20.35	130.89	61	-35.24	190.32	1534.00	0.095	0.585	-0.18	-0.37	0.03
Architex (Total Speed)	60	-62.33	65.19	55	-91.70	216.87	1683.00	0.856	0.856	0.02	-0.19	0.23
Architex (Total Score)	60	-0.14	2.84	55	-0.67	3.20	1778.50	0.472	0.661	0.08	-0.13	0.28
Anxiety (STAI)	63	-3.89	8.93	63	-3.67	8.07	1829.00	0.449	0.661	-0.08	-0.27	0.12
Arousal	63	-0.29	2.07	63	0.06	2.87	1875.50	0.592	0.691	-0.06	-0.25	0.15
Valence	63	0.71	2.42	63	-0.05	2.63	2263.50	0.167	0.585	0.14	-0.06	0.33
POMS	63	-10.21	15.40	63	-8.95	20.05	1772.00	0.301	0.661	-0.11	-0.30	0.10

Table 2F. Nonparametric t-tests (Mann-Whitney U) between the MindGym and VR groups. ‘DV’ refers to dependent variable. ‘n’ refers to group sample size. ‘ΔM’ and ‘SD’ refer to mean and standard deviation of the difference score (post – pre). ‘p.raw’ and ‘p.fdr’ refer to the uncorrected p value and FDR-corrected p value. ‘RBC’ refers to rank biserial correlation as a measure of effect size (ranging from -1 to 1) and reflects the relative proportions of positive and negative ranks. ‘RBC (low)’ and ‘RBC (high)’ refer to the lower and upper bounds of the 95% confidence interval of RBC.
