# Supplementary material for: Contrasting cognitive, behavioral, and physiological responses to breathwork vs. naturalistic stimuli in reflective chamber and VR headset environments
Source: PLOS Ment Health. 2025 Mar 12;2(3):e0000269. doi: 10.1371/journal.pmen.0000269 (PMC12798627; doi:10.1371/journal.pmen.0000269)
Supplement: S3 Table — A. MindGym Breathwork vs MindGym Rain. B. MindGym Breathwork vs VR Breathwork. C. MindGym Rain vs VR Rain. D. VR Breathwork vs VR Rain. E. Breathwork vs Rain. F. MindGym vs VR. (PDF) [file pmen.0000269.s003.pdf]

S3: Group differences in Post variables

Table 3A (MindGym Breathwork vs MindGym Rain)

	MindGym (Breathwork)			MindGym (Rain)			Mann-Whitney U test					
**DV**	**n**	**M**	**SD**	**n**	**M**	**SD**	**W**	**p.raw**	**p.fdr**	**RBC**	**RBC (low)**	**RBC (high)**
Awe	31	118.48	34.24	32	113.94	35.23	543.50	0.518	0.637	0.10	-0.27	0.44
BSE	31	4.16	1.64	32	4.28	1.73	477.00	0.795	0.795	-0.04	-0.39	0.33
EDI	28	4.21	2.11	30	3.54	2.40	505.00	0.189	0.548	0.20	-0.19	0.54
Immersion	31	67.23	11.95	32	62.78	15.21	572.00	0.299	0.548	0.15	-0.22	0.49
IPQ (General)	31	0.68	1.72	32	0.91	1.73	450.00	0.521	0.637	-0.09	-0.44	0.28
IPQ (Spatial Presence)	31	3.03	3.64	32	2.06	3.56	582.50	0.234	0.548	0.17	-0.20	0.50
IPQ (Involvement)	31	4.16	3.45	32	1.97	4.13	633.00	0.059	0.336	0.28	-0.09	0.58
IPQ (Experience Realism)	31	-2.68	3.26	32	-1.91	3.75	417.50	0.280	0.548	-0.16	-0.49	0.22
Motion (Overall)	31	19.00	10.18	32	17.80	10.69	527.00	0.674	0.741	0.06	-0.31	0.41
Toronto (Curiosity)	31	13.84	5.79	32	16.59	4.26	359.50	0.061	0.336	-0.28	-0.58	0.10
Toronto (Decentering)	31	16.65	5.96	32	16.09	4.04	549.00	0.469	0.637	0.11	-0.26	0.45

Table 3A. Nonparametric t-tests (Mann-Whitney U) between the MindGym (Breathwork) and MindGym (Rain) groups. 'DV' refers to dependent variable. 'n' refers to group sample size. 'M' and 'SD' refer to mean and standard deviation. 'p.raw' and 'p.fdr' refer to the uncorrected p value and FDR-corrected p value. 'RBC' refers to rank biserial correlation as a measure of effect size (ranging from -1 to 1) and reflects the relative proportions of positive and negative ranks. 'RBC (low)' and 'RBC (high)' refer to the lower and upper bounds of the 95% confidence interval of RBC.

Table 3B (MindGym Breathwork vs VR Breathwork)

	MindGym (Breathwork)			VR (Breathwork)			Mann-Whitney U test					
**DV**	**n**	**M**	**SD**	**n**	**M**	**SD**	**W**	**p.raw**	**p.fdr**	**RBC**	**RBC (low)**	**RBC (high)**
Awe	31	118.48	34.24	33	105.76	34.46	632.00	0.107	0.782	0.24	-0.13	0.55
BSE	31	4.16	1.64	33	3.91	1.51	553.50	0.569	0.782	0.08	-0.28	0.43
EDI	28	4.21	2.11	27	3.89	2.24	414.50	0.544	0.782	0.10	-0.30	0.46
Immersion	31	67.23	11.95	33	64.21	12.61	574.00	0.405	0.782	0.12	-0.25	0.46
IPQ (General)	31	0.68	1.72	33	0.97	1.81	463.00	0.510	0.782	-0.10	-0.44	0.27
IPQ (Spatial Presence)	31	3.03	3.64	33	3.06	3.17	517.00	0.946	0.946	0.00	-0.36	0.36
IPQ (Involvement)	31	4.16	3.45	33	3.15	4.19	582.50	0.341	0.782	0.14	-0.23	0.47
IPQ (Experience Realism)	31	-2.68	3.26	33	-2.61	3.31	544.50	0.660	0.807	0.07	-0.30	0.41
Motion (Overall)	31	19.00	10.18	33	16.81	11.08	600.50	0.232	0.782	0.17	-0.20	0.50
Toronto (Curiosity)	31	13.84	5.79	33	14.88	6.31	464.50	0.532	0.782	-0.09	-0.44	0.28
Toronto (Decentering)	31	16.65	5.96	33	17.18	4.48	506.00	0.946	0.946	-0.01	-0.37	0.35

Table 3B. Nonparametric t-tests (Mann-Whitney U) between the MindGym (Breathwork) and VR (Breathwork) groups. 'DV' refers to dependent variable. 'n' refers to group sample size. 'M' and 'SD' refer to mean and standard deviation. 'p.raw' and 'p.fdr' refer to the uncorrected p value and FDR-corrected p value. 'RBC' refers to rank biserial correlation as a measure of effect size (ranging from -1 to 1) and reflects the relative proportions of positive and negative ranks. 'RBC (low)' and 'RBC (high)' refer to the lower and upper bounds of the 95% confidence interval of RBC.

Table 3C (MindGym Rain vs VR Rain)

	MindGym (Rain)			VR (Rain)			Mann-Whitney U test					
DV	n	M	SD	n	M	SD	W	p.raw	p.fdr	RBC	RBC (low)	RBC (high)
Awe	32	113.94	35.23	30	114.83	29.69	478.50	0.989	0.989	0.00	-0.37	0.36
BSE	32	4.28	1.73	30	4.10	1.67	515.00	0.620	0.950	0.07	-0.30	0.43
EDI	30	3.54	2.40	27	3.35	2.67	430.50	0.689	0.950	0.06	-0.32	0.43
Immersion	32	62.78	15.21	30	61.97	10.71	525.00	0.530	0.950	0.09	-0.28	0.44
IPQ (General)	32	0.91	1.73	30	1.17	1.34	453.50	0.706	0.950	-0.06	-0.41	0.32
IPQ (Spatial Presence)	32	2.06	3.56	30	3.23	3.77	380.50	0.161	0.886	-0.21	-0.53	0.17
IPQ (Involvement)	32	1.97	4.13	30	1.33	4.11	559.00	0.267	0.950	0.17	-0.21	0.50
IPQ (Experience Realism)	32	-1.91	3.75	30	-3.13	2.97	629.00	0.035	0.385	0.31	-0.06	0.61
Motion (Overall)	32	17.80	10.69	30	16.83	6.68	473.00	0.927	0.989	-0.02	-0.38	0.35
Toronto (Curiosity)	32	16.59	4.26	30	16.60	6.32	459.50	0.777	0.950	-0.04	-0.40	0.33
Toronto (Decentering)	32	16.09	4.04	30	16.87	5.79	423.00	0.424	0.950	-0.12	-0.46	0.26

Table 3C. Nonparametric t-tests (Mann-Whitney U) between the MindGym (Rain) and VR (Rain) groups. 'DV' refers to dependent variable. 'n' refers to group sample size. 'M' and 'SD' refer to mean and standard deviation. 'p.raw' and 'p.fdr' refer to the uncorrected p value and FDR-corrected p value. 'RBC' refers to rank biserial correlation as a measure of effect size (ranging from -1 to 1) and reflects the relative proportions of positive and negative ranks. 'RBC (low)' and 'RBC (high)' refer to the lower and upper bounds of the 95% confidence interval of RBC.

Table 3D (VR Breathwork vs VR Rain)

	VR (Breathwork)			VR (Rain)			Mann-Whitney U test					
DV	n	M	SD	n	M	SD	W	p.raw	p.fdr	RBC	RBC (low)	RBC (high)
Awe	33	105.76	34.46	30	114.83	29.69	408.00	0.234	0.673	-0.18	-0.51	0.20
BSE	33	3.91	1.51	30	4.10	1.67	467.00	0.699	0.879	-0.06	-0.41	0.31
EDI	27	3.89	2.24	27	3.35	2.67	406.00	0.478	0.751	0.11	-0.29	0.48
Immersion	33	64.21	12.61	30	61.97	10.71	561.00	0.367	0.673	0.13	-0.24	0.47
IPQ (General)	33	0.97	1.81	30	1.17	1.34	479.50	0.832	0.915	-0.03	-0.39	0.33
IPQ (Spatial Presence)	33	3.06	3.17	30	3.23	3.77	468.50	0.719	0.879	-0.05	-0.41	0.31
IPQ (Involvement)	33	3.15	4.19	30	1.33	4.11	620.00	0.086	0.673	0.25	-0.12	0.56
IPQ (Experience Realism)	33	-2.61	3.31	30	-3.13	2.97	564.00	0.343	0.673	0.14	-0.23	0.48
Motion (Overall)	33	16.81	11.08	30	16.83	6.68	421.50	0.313	0.673	-0.15	-0.48	0.23
Toronto (Curiosity)	33	14.88	6.31	30	16.60	6.32	414.00	0.267	0.673	-0.16	-0.50	0.21
Toronto (Decentering)	33	17.18	4.48	30	16.87	5.79	493.50	0.989	0.989	0.00	-0.36	0.36

Table 3D. Nonparametric t-tests (Mann-Whitney U) between the VR (Breathwork) and VR (Rain) groups. 'DV' refers to dependent variable. 'n' refers to group sample size. 'M' and 'SD' refer to mean and standard deviation. 'p.raw' and 'p.fdr' refer to the uncorrected p value and FDR-corrected p value. 'RBC' refers to rank biserial correlation as a measure of effect size (ranging from -1 to 1) and reflects the relative proportions of positive and negative ranks. 'RBC (low)' and 'RBC (high)' refer to the lower and upper bounds of the 95% confidence interval of RBC.

Table 3E (Breathwork vs Rain)

	Breathwork			Rain			Mann-Whitney U test					
**DV**	**n**	**M**	**SD**	**n**	**M**	**SD**	**W**	**p.raw**	**p.fdr**	**RBC**	**RBC (low)**	**RBC (high)**
Awe	64	111.92	34.68	62	114.37	32.41	1928.50	0.788	0.854	-0.03	-0.29	0.23
BSE	64	4.03	1.56	62	4.19	1.69	1877.50	0.598	0.787	-0.05	-0.31	0.21
EDI	55	4.05	2.16	57	3.45	2.51	1818.00	0.146	0.501	0.16	-0.12	0.42
Immersion	64	65.67	12.29	62	62.39	13.12	2258.00	0.182	0.501	0.14	-0.13	0.38
IPQ (General)	64	0.83	1.76	62	1.03	1.55	1859.50	0.535	0.787	-0.06	-0.32	0.20
IPQ (Spatial Presence)	64	3.05	3.38	62	2.63	3.68	2115.00	0.522	0.787	0.07	-0.20	0.32
IPQ (Involvement)	64	3.64	3.85	62	1.66	4.10	2501.50	0.011	0.121	0.26	0.00	0.49
IPQ (Experience Realism)	64	-2.64	3.26	62	-2.50	3.42	1946.00	0.854	0.854	-0.02	-0.28	0.24
Motion (Overall)	64	17.87	10.62	62	17.33	8.92	1889.00	0.644	0.787	-0.05	-0.30	0.22
Toronto (Curiosity)	64	14.38	6.04	62	16.60	5.31	1582.00	0.050	0.275	-0.20	-0.44	0.06
Toronto (Decentering)	64	16.92	5.22	62	16.47	4.94	2096.50	0.584	0.787	0.06	-0.21	0.31

Table 3E. Nonparametric t-tests (Mann-Whitney U) between the Breathwork and Rain groups. ‘DV’ refers to dependent variable. ‘n’ refers to group sample size. ‘M’ and ‘SD’ refer to mean and standard deviation. ‘p.raw’ and ‘p.fdr’ refer to the uncorrected p value and FDR-corrected p value. ‘RBC’ refers to rank biserial correlation as a measure of effect size (ranging from -1 to 1) and reflects the relative proportions of positive and negative ranks. ‘RBC (low)’ and ‘RBC (high)’ refer to the lower and upper bounds of the 95% confidence interval of RBC.

Table 3F (MindGym vs VR)

	MindGym			VR			Mann-Whitney U test					
**DV**	**n**	**M**	**SD**	**n**	**M**	**SD**	**W**	**p.raw**	**p.fdr**	**RBC**	**RBC (low)**	**RBC (high)**
Awe	63	116.18	34.54	63	110.08	32.34	2236.50	0.220	0.586	0.13	-0.14	0.38
BSE	63	4.22	1.67	63	4.00	1.58	2140.00	0.440	0.586	0.08	-0.19	0.33
EDI	58	3.86	2.27	54	3.62	2.45	1664.00	0.570	0.586	0.06	-0.22	0.33
Immersion	63	64.97	13.78	63	63.14	11.71	2210.00	0.272	0.586	0.11	-0.15	0.36
IPQ (General)	63	0.79	1.72	63	1.06	1.60	1819.50	0.410	0.586	-0.08	-0.34	0.18
IPQ (Spatial Presence)	63	2.54	3.60	63	3.14	3.44	1783.50	0.326	0.586	-0.10	-0.35	0.16
IPQ (Involvement)	63	3.05	3.94	63	2.29	4.22	2250.00	0.194	0.586	0.13	-0.13	0.38
IPQ (Experience Realism)	63	-2.29	3.51	63	-2.86	3.14	2359.00	0.067	0.586	0.19	-0.08	0.43
Motion (Overall)	63	18.39	10.37	63	16.82	9.17	2162.00	0.386	0.586	0.09	-0.18	0.34
Toronto (Curiosity)	63	15.24	5.21	63	15.70	6.33	1872.50	0.586	0.586	-0.06	-0.31	0.21
Toronto (Decentering)	63	16.37	5.05	63	17.03	5.11	1851.00	0.515	0.586	-0.07	-0.32	0.20

Table 3F. Nonparametric t-tests (Mann-Whitney U) between the MindGym and VR groups. ‘DV’ refers to dependent variable. ‘n’ refers to group sample size. ‘M’ and ‘SD’ refer to mean and standard deviation. ‘p.raw’ and ‘p.fdr’ refer to the uncorrected p value and FDR-corrected p value. ‘RBC’ refers to rank biserial correlation as a measure of effect size (ranging from -1 to 1) and reflects the relative proportions of positive and negative ranks. ‘RBC (low)’ and ‘RBC (high)’ refer to the lower and upper bounds of the 95% confidence interval of RBC.
