# Supplementary material for: Contrasting cognitive, behavioral, and physiological responses to breathwork vs. naturalistic stimuli in reflective chamber and VR headset environments
Source: PLOS Ment Health. 2025 Mar 12;2(3):e0000269. doi: 10.1371/journal.pmen.0000269 (PMC12798627; doi:10.1371/journal.pmen.0000269)
Supplement: S5 Table — A. Architex Total Score. B. Architex Total Speed. C. TMT TRACC. D. Arousal. E. POMS. F. STAI. G. Valence. H. Awe. I. EDI. J. BSE. (PDF) [file pmen.0000269.s005.pdf]

S5: Moderation Tables

Table 5A (Architex Total Score)

DV	Tech x Stim x Moderator			Tech x Moderator			Stim x Moderator			Moderator				
Architex (Total Score)	**F**	**p.raw**	**p.fdr**	**F**	**p.raw**	**p.fdr**	**F**	**p.raw**	**p.fdr**	**b**	**SE**	**Z**	**p.raw**	**p.fdr**
**Psych Moderator**														
DPES	0.28	0.598	0.941	0.98	0.322	0.661	5.68	0.017	0.128	0.15	0.28	0.55	0.581	0.846
Immersion	5.71	0.017	0.254	1.10	0.295	0.661	0.07	0.795	0.951	-0.01	0.02	-0.45	0.652	0.846
IPQ (General)	2.09	0.148	0.452	0.00	0.996	0.996	0.37	0.543	0.905	0.04	0.14	0.30	0.761	0.846
IPQ (Involvement)	3.29	0.070	0.380	0.47	0.492	0.671	0.16	0.690	0.941	-0.01	0.06	-0.20	0.846	0.846
IPQ (Spatial Presence)	0.01	0.908	0.941	2.34	0.126	0.629	0.02	0.903	0.968	0.02	0.07	0.24	0.808	0.846
IPQ (Experienced Realism)	1.37	0.242	0.560	0.04	0.834	0.958	0.44	0.509	0.905	0.01	0.07	0.20	0.844	0.846
MODTAS	0.09	0.764	0.941	0.66	0.418	0.661	1.50	0.221	0.827	0.00	0.01	0.38	0.708	0.846
Openness	2.06	0.151	0.452	0.02	0.894	0.958	5.74	0.017	0.128	-0.01	0.07	-0.20	0.839	0.846
**Physio Moderator**														
Motion (overall)	0.22	0.639	0.941	3.55	0.059	0.446	0.00	0.968	0.968	0.04	0.04	1.03	0.305	0.819
Breath Rate (first 2 min)	0.03	0.860	0.941	0.72	0.396	0.661	0.19	0.662	0.941	-0.08	0.07	-1.09	0.276	0.819
HR (first 1 min)	3.15	0.076	0.380	0.59	0.441	0.661	4.67	0.031	0.154	-0.02	0.02	-0.98	0.326	0.819
HRV (first 5 min)	1.26	0.261	0.560	1.08	0.298	0.661	0.45	0.502	0.905	4.36	4.46	0.98	0.327	0.819
Breath Rate change (last - first)	0.01	0.941	0.941	0.22	0.642	0.803	0.60	0.439	0.905	0.06	0.06	1.11	0.266	0.819
HR change (last - first)	0.68	0.409	0.767	10.08	0.002	0.023	0.05	0.824	0.951	-0.12	0.06	-1.95	0.051	0.762
HRV change (last - first)	0.04	0.852	0.941	0.78	0.379	0.661	0.55	0.457	0.905	-0.95	3.62	-0.26	0.793	0.846

Table 5A. Results from GLM tests of moderation. ‘DV’ refers to dependent variable of Architex (Total Score) calculated as a difference score (post – pre). ‘Psych Moderator’ and ‘Physio Moderator’ refer to either the psychological or physiological variable defined as the moderator. ‘Tech x Stim x Moderator’ refers to the model of a three-way interaction between the moderator and the group factors of Tech (MindGym vs VR) and Stimuli (Breathwork vs Rain). ‘Tech x Moderator’ refers to the model of a two-way interaction between the moderator and the group factor of Tech (MindGym vs VR). ‘Stim x Moderator’ refers to the model of a two-way interaction between the moderator and the group factor of Stimuli (Breathwork vs Rain). ‘Moderator’ refers to the effect of the moderator from the model of only main effects. ‘F’ refers to F-test from the GLM model. ‘b’ and ‘SE’ refer to the unstandardized beta coefficient and its standard error. ‘Z’ refers to the Z-test from the model. ‘p.raw’ and ‘p.fdr’ refer to the uncorrected p value and FDR-corrected p value.

Table 5B (Architex Total Speed)

DV	Tech x Stim x Moderator			Tech x Moderator			Stim x Moderator			Moderator				
Architex (Total Speed)	**F**	**p.raw**	**p.fdr**	**F**	**p.raw**	**p.fdr**	**F**	**p.raw**	**p.fdr**	**b**	**SE**	**Z**	**p.raw**	**p.fdr**
**Psych Moderator**														
DPES	0.70	0.403	0.792	0.50	0.481	0.656	0.08	0.776	0.984	-8.68	6.53	-1.33	0.184	0.824
Immersion	0.02	0.887	0.887	3.05	0.081	0.394	0.02	0.881	0.984	0.54	0.49	1.09	0.274	0.824
IPQ (General)	0.18	0.669	0.837	1.32	0.251	0.470	0.18	0.669	0.984	2.67	3.46	0.77	0.440	0.824
IPQ (Involvement)	0.09	0.764	0.881	1.77	0.184	0.394	0.54	0.463	0.984	2.10	1.57	1.33	0.183	0.824
IPQ (Spatial Presence)	0.03	0.854	0.887	0.09	0.761	0.902	0.09	0.760	0.984	-1.15	1.87	-0.61	0.540	0.843
IPQ (Experienced Realism)	0.19	0.664	0.837	2.11	0.147	0.394	0.01	0.946	0.984	0.95	1.63	0.58	0.562	0.843
MODTAS	0.69	0.407	0.792	2.39	0.122	0.394	0.02	0.893	0.984	-0.02	0.22	-0.08	0.933	0.980

Openness	2.21	0.137	0.792	0.08	0.782	0.902	0.00	0.984	0.984	-1.48	1.39	-1.06	0.289	0.824
**Physio Moderator**														
Motion (overall)	0.74	0.390	0.792	1.91	0.167	0.394	6.83	0.009	0.068	-0.10	1.01	-0.10	0.924	0.980
Breath Rate (first 2 min)	0.94	0.332	0.792	0.65	0.419	0.628	0.53	0.465	0.984	0.55	1.86	0.30	0.767	0.980
HR (first 1 min)	0.34	0.559	0.837	1.87	0.171	0.394	1.03	0.311	0.984	0.55	0.66	0.84	0.403	0.824
HRV (first 5 min)	2.07	0.156	0.792	6.50	0.013	0.195	8.49	0.005	0.068	344.90	132.69	2.60	0.009	0.140
Breath Rate change (last - first)	0.41	0.522	0.837	0.01	0.906	0.906	0.96	0.328	0.984	1.41	1.56	0.91	0.365	0.824
HR change (last - first)	0.64	0.423	0.792	0.04	0.848	0.906	0.03	0.863	0.984	0.04	1.57	0.03	0.980	0.980
HRV change (last - first)	65.00	0.300	0.792	68.00	0.386	0.628	68.00	0.676	0.984	9.84	82.18	0.12	0.905	0.980

Table 5B. Results from GLM tests of moderation. 'DV' refers to dependent variable of Architex (Total Speed) calculated as a difference score (post – pre). 'Psych Moderator' and 'Physio Moderator' refer to either the psychological or physiological variable defined as the moderator. 'Tech x Stim x Moderator' refers to the model of a three-way interaction between the moderator and the group factors of Tech (MindGym vs VR) and Stimuli (Breathwork vs Rain). 'Tech x Moderator' refers to the model of a two-way interaction between the moderator and the group factor of Tech (MindGym vs VR). 'Stim x Moderator' refers to the model of a two-way interaction between the moderator and the group factor of Stimuli (Breathwork vs Rain). 'Moderator' refers to the effect of the moderator from the model of only main effects. 'F' refers to F-test from the GLM model. 'b' and 'SE' refer to the unstandardized beta coefficient and its standard error. 'Z' refers to the Z-test from the model. 'p.raw' and 'p.fdr' refer to the uncorrected p value and FDR-corrected p value.

Table 5C (TMT RTACC)

**DV**	**Tech x Stim x Moderator**			**Tech x Moderator**			**Stim x Moderator**			**Moderator**				
TMT (RTACC)	**F**	**p.raw**	**p.fdr**	**F**	**p.raw**	**p.fdr**	**F**	**p.raw**	**p.fdr**	**b**	**SE**	**Z**	**p.raw**	**p.fdr**
**Psych Moderator**														
DPES	0.72	0.397	0.725	0.27	0.601	0.772	0.31	0.581	0.686	-7.74	4.33	-1.79	0.074	0.335
Immersion	0.04	0.850	0.850	0.30	0.582	0.772	4.17	0.041	0.529	0.25	0.27	0.92	0.357	0.669
IPQ (General)	1.84	0.175	0.609	0.02	0.895	0.895	0.54	0.463	0.686	1.77	2.16	0.82	0.414	0.689
IPQ (Involvement)	6.66	0.010	0.149	0.22	0.642	0.772	0.28	0.594	0.686	1.23	0.87	1.41	0.157	0.473
IPQ (Spatial Presence)	0.19	0.664	0.850	0.46	0.496	0.772	0.53	0.465	0.686	1.40	1.22	1.15	0.252	0.612
IPQ (Experienced Realism)	1.22	0.270	0.609	2.63	0.105	0.759	1.39	0.238	0.595	2.08	1.06	1.96	0.050	0.335
MODTAS	0.24	0.624	0.850	0.77	0.380	0.772	0.33	0.564	0.686	-0.23	0.13	-1.70	0.089	0.335
Openness	0.61	0.435	0.725	0.18	0.673	0.772	0.00	0.998	0.998	0.15	0.99	0.15	0.878	0.940
**Physio Moderator**														
Motion (overall)	0.06	0.811	0.850	1.63	0.202	0.759	2.74	0.098	0.529	-0.31	0.49	-0.63	0.528	0.720
Breath Rate (first 2 min)	1.15	0.284	0.609	0.13	0.720	0.772	1.25	0.264	0.595	0.93	0.87	1.07	0.286	0.612
HR (first 1 min)	0.12	0.728	0.850	0.13	0.720	0.772	1.26	0.262	0.595	0.12	0.30	0.39	0.695	0.869
HRV (first 5 min)	3.41	0.065	0.324	3.62	0.057	0.759	0.44	0.505	0.686	131.00	53.94	2.43	0.015	0.227
Breath Rate change (last - first)	1.35	0.245	0.609	0.86	0.354	0.772	2.62	0.106	0.529	-0.04	0.74	-0.05	0.957	0.957
HR change (last - first)	0.08	0.776	0.850	0.53	0.468	0.772	1.18	0.278	0.595	0.67	0.94	0.72	0.475	0.712
HRV change (last - first)	63.00	0.038	0.287	66.00	0.167	0.759	0.00	0.961	0.998	-11.11	39.32	-0.28	0.777	0.897

Table 5C. Results from GLM tests of moderation. 'DV' refers to dependent variable of TMT (RTACC) calculated as a difference score (post – pre). 'Psych Moderator' and 'Physio Moderator' refer to either the psychological or physiological variable defined as the moderator. 'Tech x Stim x Moderator' refers to the model of a three-way interaction between the moderator and the group factors of Tech (MindGym vs VR) and Stimuli (Breathwork vs Rain). 'Tech x Moderator' refers to the model of a two-way interaction between the moderator and the group factor of Tech (MindGym vs VR). 'Stim x Moderator' refers to the model of a two-way interaction between the moderator and the group factor of Stimuli (Breathwork vs Rain). 'Moderator' refers to the effect of the moderator from

the model of only main effects. ‘F’ refers to F-test from the GLM model. ‘b’ and ‘SE’ refer to the unstandardized beta coefficient and its standard error. ‘Z’ refers to the Z-test from the model. ‘p.raw’ and ‘p.fdr’ refer to the uncorrected p value and FDR-corrected p value.

Table 5D (Arousal)

DV	Tech x Stim x Moderator			Tech x Moderator			Stim x Moderator			Moderator				
Arousal	**F**	**p.raw**	**p.fdr**	**F**	**p.raw**	**p.fdr**	**F**	**p.raw**	**p.fdr**	**b**	**SE**	**Z**	**p.raw**	**p.fdr**
**Psych Moderator**														
DPES	0.42	0.518	0.948	2.29	0.130	0.456	0.00	0.983	0.983	0.01	0.25	0.04	0.970	0.980
Immersion	0.56	0.454	0.948	0.00	0.982	0.982	1.16	0.281	0.919	0.00	0.02	0.04	0.969	0.980
IPQ (General)	0.03	0.875	0.948	1.42	0.233	0.545	0.63	0.428	0.983	0.11	0.13	0.79	0.428	0.980
IPQ (Involvement)	0.82	0.366	0.948	2.05	0.152	0.456	0.02	0.877	0.983	0.01	0.06	0.16	0.870	0.980
IPQ (Spatial Presence)	0.05	0.826	0.948	0.16	0.685	0.857	3.35	0.067	0.918	-0.07	0.07	-1.04	0.298	0.980
IPQ (Experienced Realism)	1.70	0.192	0.720	1.13	0.287	0.545	0.01	0.940	0.983	0.00	0.06	0.05	0.960	0.980
MODTAS	0.63	0.426	0.948	2.14	0.144	0.456	0.03	0.854	0.983	-0.01	0.01	-0.82	0.415	0.980
Openness	1.72	0.190	0.720	2.38	0.123	0.456	1.29	0.256	0.919	-0.03	0.05	-0.52	0.606	0.980
**Physio Moderator**														
Motion (overall)	0.01	0.934	0.948	1.12	0.291	0.545	0.01	0.923	0.983	-0.01	0.03	-0.34	0.731	0.980
Breath Rate (first 2 min)	0.21	0.649	0.948	0.22	0.641	0.857	0.16	0.694	0.983	0.00	0.06	-0.03	0.980	0.980
HR (first 1 min)	0.00	0.948	0.948	0.11	0.744	0.859	0.00	0.961	0.983	0.03	0.03	1.26	0.209	0.980
HRV (first 5 min)	0.07	0.792	0.948	0.64	0.426	0.709	1.05	0.306	0.919	-2.18	5.56	-0.39	0.696	0.980
Breath Rate change (last - first)	2.61	0.106	0.720	0.01	0.935	0.982	0.05	0.825	0.983	0.02	0.05	0.43	0.670	0.980
HR change (last - first)	66.00	0.845	0.948	69.00	0.655	0.857	69.00	0.122	0.918	0.08	0.07	1.22	0.221	0.980
HRV change (last - first)	2.27	0.132	0.720	6.74	0.009	0.141	0.31	0.578	0.983	2.09	3.51	0.60	0.551	0.980

Table 5D. Results from GLM tests of moderation. ‘DV’ refers to dependent variable of Arousal calculated as a difference score (post – pre). ‘Psych Moderator’ and ‘Physio Moderator’ refer to either the psychological or physiological variable defined as the moderator. ‘Tech x Stim x Moderator’ refers to the model of a three-way interaction between the moderator and the group factors of Tech (MindGym vs VR) and Stimuli (Breathwork vs Rain). ‘Tech x Moderator’ refers to the model of a two-way interaction between the moderator and the group factor of Tech (MindGym vs VR). ‘Stim x Moderator’ refers to the model of a two-way interaction between the moderator and the group factor of Stimuli (Breathwork vs Rain). ‘Moderator’ refers to the effect of the moderator from the model of only main effects. ‘F’ refers to F-test from the GLM model. ‘b’ and ‘SE’ refer to the unstandardized beta coefficient and its standard error. ‘Z’ refers to the Z-test from the model. ‘p.raw’ and ‘p.fdr’ refer to the uncorrected p value and FDR-corrected p value.

Table 5E (POMS)

DV	Tech x Stim x Moderator			Tech x Moderator			Stim x Moderator			Moderator				
POMS	**F**	**p.raw**	**p.fdr**	**F**	**p.raw**	**p.fdr**	**F**	**p.raw**	**p.fdr**	**b**	**SE**	**Z**	**p.raw**	**p.fdr**
**Psych Moderator**														
DPES	0.05	0.831	0.891	0.74	0.390	0.824	1.62	0.206	0.806	-0.86	1.65	-0.52	0.603	0.886
Immersion	0.07	0.795	0.891	0.53	0.466	0.824	0.01	0.923	0.940	-0.04	0.11	-0.35	0.728	0.911
IPQ (General)	0.37	0.543	0.757	2.67	0.105	0.674	0.33	0.568	0.806	0.81	0.80	1.01	0.313	0.886
IPQ (Involvement)	3.05	0.084	0.599	0.95	0.331	0.824	0.29	0.591	0.806	-0.04	0.35	-0.10	0.918	0.918
IPQ (Spatial Presence)	2.32	0.131	0.599	0.59	0.445	0.824	0.33	0.569	0.806	-0.24	0.43	-0.55	0.581	0.886
IPQ (Experienced Realism)	0.55	0.462	0.757	0.01	0.933	0.933	0.02	0.892	0.940	-0.18	0.39	-0.46	0.649	0.886
MODTAS	0.80	0.374	0.701	2.35	0.128	0.674	0.21	0.645	0.806	-0.01	0.05	-0.16	0.871	0.918

Openness	0.80	0.373	0.701	0.24	0.628	0.824	0.30	0.588	0.806	-0.22	0.37	-0.60	0.551	0.886
**Physio Moderator**														
Motion (overall)	0.06	0.808	0.891	0.13	0.721	0.824	1.05	0.307	0.806	0.42	0.22	1.93	0.053	0.398
Breath Rate (first 2 min)	1.26	0.265	0.662	0.18	0.669	0.824	1.28	0.261	0.806	-0.42	0.41	-1.01	0.311	0.886
HR (first 1 min)	2.23	0.140	0.599	0.12	0.732	0.824	0.28	0.597	0.806	-0.03	0.15	-0.18	0.855	0.918
HRV (first 5 min)	0.01	0.934	0.934	0.82	0.368	0.824	0.01	0.940	0.940	21.22	33.79	0.63	0.530	0.886
Breath Rate change (last - first)	1.68	0.200	0.599	0.19	0.661	0.824	0.30	0.584	0.806	0.45	0.32	1.38	0.169	0.843
HR change (last - first)	1.77	0.184	0.599	2.24	0.135	0.674	2.32	0.128	0.806	-0.22	0.41	-0.52	0.601	0.886
HRV change (last - first)	0.35	0.555	0.757	0.09	0.769	0.824	0.74	0.391	0.806	-38.21	18.29	-2.09	0.037	0.398

Table 5E. Results from GLM tests of moderation. 'DV' refers to dependent variable of POMS (Total mood disturbance) calculated as a difference score (post – pre). 'Psych Moderator' and 'Physio Moderator' refer to either the psychological or physiological variable defined as the moderator. 'Tech x Stim x Moderator' refers to the model of a three-way interaction between the moderator and the group factors of Tech (MindGym vs VR) and Stimuli (Breathwork vs Rain). 'Tech x Moderator' refers to the model of a two-way interaction between the moderator and the group factor of Tech (MindGym vs VR). 'Stim x Moderator' refers to the model of a two-way interaction between the moderator and the group factor of Stimuli (Breathwork vs Rain). 'Moderator' refers to the effect of the moderator from the model of only main effects. 'F' refers to F-test from the GLM model. 'b' and 'SE' refer to the unstandardized beta coefficient and its standard error. 'Z' refers to the Z-test from the model. 'p.raw' and 'p.fdr' refer to the uncorrected p value and FDR-corrected p value.

Table 5F (STAI)

**DV**	**Tech x Stim x Moderator**			**Tech x Moderator**			**Stim x Moderator**			**Moderator**				
Anxiety (STAI)	**F**	**p.raw**	**p.fdr**	**F**	**p.raw**	**p.fdr**	**F**	**p.raw**	**p.fdr**	**b**	**SE**	**Z**	**p.raw**	**p.fdr**
**Psych Moderator**														
DPES	0.04	0.842	0.985	1.98	0.162	0.608	2.71	0.103	0.411	-0.35	0.76	-0.46	0.647	0.814
Immersion	0.53	0.467	0.985	0.37	0.545	0.817	0.99	0.322	0.411	-0.05	0.05	-1.01	0.313	0.740
IPQ (General)	0.00	0.963	0.985	2.00	0.160	0.608	1.20	0.275	0.411	-0.42	0.37	-1.16	0.247	0.740
IPQ (Involvement)	0.03	0.858	0.985	0.12	0.729	0.831	0.17	0.681	0.681	0.03	0.16	0.17	0.867	0.929
IPQ (Spatial Presence)	0.34	0.564	0.985	2.37	0.127	0.608	0.25	0.621	0.681	-0.16	0.19	-0.83	0.407	0.740
IPQ (Experienced Realism)	0.02	0.876	0.985	1.09	0.299	0.699	1.15	0.285	0.411	-0.12	0.18	-0.69	0.493	0.740
MODTAS	0.10	0.758	0.985	0.62	0.432	0.720	6.42	0.013	0.095	-0.04	0.02	-1.67	0.095	0.357
Openness	0.58	0.450	0.985	0.97	0.327	0.699	9.58	0.003	0.038	-0.27	0.16	-1.68	0.093	0.357
**Physio Moderator**														
Motion (overall)	1.45	0.232	0.985	5.72	0.018	0.276	1.43	0.235	0.411	0.07	0.10	0.72	0.470	0.740
Breath Rate (first 2 min)	0.05	0.817	0.985	0.08	0.785	0.831	1.81	0.182	0.411	-0.56	0.19	-2.90	0.004	0.057
HR (first 1 min)	0.01	0.915	0.985	0.12	0.732	0.831	0.23	0.636	0.681	0.00	0.08	-0.04	0.969	0.969
HRV (first 5 min)	0.00	0.985	0.985	0.05	0.831	0.831	0.97	0.329	0.411	-6.19	16.37	-0.38	0.705	0.814
Breath Rate change (last - first)	2.20	0.143	0.985	0.21	0.646	0.831	2.40	0.126	0.411	0.39	0.15	2.50	0.013	0.094
HR change (last - first)	0.51	0.479	0.985	0.81	0.373	0.699	1.88	0.175	0.411	-0.15	0.20	-0.76	0.446	0.740
HRV change (last - first)	0.06	0.815	0.985	1.33	0.253	0.699	1.25	0.268	0.411	3.98	9.53	0.42	0.677	0.814

Table 5F. Results from GLM tests of moderation. 'DV' refers to dependent variable of STAI calculated as a difference score (post – pre). 'Psych Moderator' and 'Physio Moderator' refer to either the psychological or physiological variable defined as the moderator. 'Tech x Stim x Moderator' refers to the model of a three-way interaction between the moderator and the group factors of Tech (MindGym vs VR) and Stimuli (Breathwork vs Rain). 'Tech x Moderator' refers to the model of a two-way interaction between the moderator and the group factor of Tech (MindGym vs VR). 'Stim x Moderator' refers to the model of a two-way interaction between

the moderator and the group factor of Stimuli (Breathwork vs Rain). 'Moderator' refers to the effect of the moderator from the model of only main effects. 'F' refers to F-test from the GLM model. 'b' and 'SE' refer to the unstandardized beta coefficient and its standard error. 'Z' refers to the Z-test from the model. 'p.raw' and 'p.fdr' refer to the uncorrected p value and FDR-corrected p value.

Table 5G (Valence)

DV	Tech x Stim x Moderator			Tech x Moderator			Stim x Moderator			Moderator				
Valence	**F**	**p.raw**	**p.fdr**	**F**	**p.raw**	**p.fdr**	**F**	**p.raw**	**p.fdr**	**b**	**SE**	**Z**	**p.raw**	**p.fdr**
**Psych Moderator**														
DPES	0.02	0.894	0.915	0.34	0.563	0.876	0.12	0.733	0.909	-0.07	0.20	-0.33	0.742	0.823
Immersion	1.48	0.227	0.710	0.10	0.759	0.876	0.01	0.909	0.909	-0.01	0.01	-0.99	0.323	0.813
IPQ (General)	0.09	0.769	0.915	2.50	0.117	0.876	0.47	0.496	0.909	-0.05	0.10	-0.45	0.651	0.813
IPQ (Involvement)	3.46	0.066	0.583	1.82	0.181	0.876	0.08	0.775	0.909	0.00	0.04	0.01	0.995	0.995
IPQ (Spatial Presence)	0.01	0.915	0.915	0.10	0.748	0.876	0.69	0.407	0.909	0.03	0.05	0.50	0.619	0.813
IPQ (Experienced Realism)	0.21	0.652	0.915	0.19	0.663	0.876	0.46	0.501	0.909	0.03	0.05	0.63	0.526	0.813
MODTAS	0.10	0.758	0.915	1.48	0.226	0.876	1.17	0.283	0.909	0.00	0.01	-0.30	0.768	0.823
Openness	0.32	0.574	0.915	0.56	0.458	0.876	0.76	0.385	0.909	0.05	0.05	1.04	0.299	0.813
**Physio Moderator**														
Motion (overall)	0.65	0.421	0.807	1.38	0.244	0.876	1.34	0.249	0.909	-0.05	0.02	-1.93	0.054	0.402
Breath Rate (first 2 min)	0.63	0.431	0.807	0.00	0.987	0.987	0.90	0.346	0.909	-0.03	0.05	-0.51	0.607	0.813
HR (first 1 min)	0.02	0.903	0.915	0.11	0.743	0.876	0.30	0.588	0.909	0.01	0.02	0.51	0.611	0.813
HRV (first 5 min)	3.22	0.078	0.583	0.01	0.937	0.987	0.04	0.836	0.909	11.13	4.24	2.62	0.009	0.131
Breath Rate change (last - first)	1.54	0.220	0.710	0.77	0.384	0.876	0.13	0.715	0.909	-0.02	0.04	-0.60	0.551	0.813
HR change (last - first)	0.91	0.343	0.807	1.11	0.296	0.876	0.02	0.898	0.909	0.03	0.04	0.62	0.536	0.813
HRV change (last - first)	1.43	0.237	0.710	0.13	0.725	0.876	0.10	0.756	0.909	-3.21	2.47	-1.30	0.194	0.813

Table 5G. Results from GLM tests of moderation. 'DV' refers to dependent variable of Valence calculated as a difference score (post – pre). 'Psych Moderator' and 'Physio Moderator' refer to either the psychological or physiological variable defined as the moderator. 'Tech x Stim x Moderator' refers to the model of a three-way interaction between the moderator and the group factors of Tech (MindGym vs VR) and Stimuli (Breathwork vs Rain). 'Tech x Moderator' refers to the model of a two-way interaction between the moderator and the group factor of Tech (MindGym vs VR). 'Stim x Moderator' refers to the model of a two-way interaction between the moderator and the group factor of Stimuli (Breathwork vs Rain). 'Moderator' refers to the effect of the moderator from the model of only main effects. 'F' refers to F-test from the GLM model. 'b' and 'SE' refer to the unstandardized beta coefficient and its standard error. 'Z' refers to the Z-test from the model. 'p.raw' and 'p.fdr' refer to the uncorrected p value and FDR-corrected p value.

Table 5H (Awe)

DV	Tech x Stim x Moderator			Tech x Moderator			Stim x Moderator			Moderator				
Awe	**F**	**p.raw**	**p.fdr**	**F**	**p.raw**	**p.fdr**	**F**	**p.raw**	**p.fdr**	**b**	**SE**	**Z**	**p.raw**	**p.fdr**
**Psych Moderator**														
DPES	1.74	0.190	0.824	0.14	0.711	0.887	0.20	0.657	0.957	13.89	3.48	4.00	< .0001	< .001
Immersion	0.03	0.870	0.892	0.08	0.776	0.887	0.02	0.896	0.957	1.16	0.22	5.17	< .0001	< .001
IPQ (General)	0.02	0.892	0.892	4.70	0.032	0.362	1.92	0.169	0.853	6.18	1.72	3.59	< .001	0.002
IPQ (Involvement)	1.08	0.301	0.892	0.12	0.727	0.887	0.82	0.367	0.853	2.24	0.76	2.96	0.003	0.009
IPQ (Spatial Presence)	0.38	0.538	0.892	0.00	0.957	0.957	0.77	0.381	0.853	1.83	0.93	1.98	0.048	0.104
IPQ (Experienced Realism)	1.52	0.220	0.824	0.34	0.560	0.887	3.98	0.048	0.725	0.68	0.87	0.78	0.436	0.620

MODTAS	0.56	0.458	0.892	0.05	0.828	0.887	0.38	0.539	0.899	0.37	0.11	3.28	0.001	0.004
Openness	0.36	0.553	0.892	0.85	0.359	0.796	0.03	0.871	0.957	1.22	0.81	1.51	0.131	0.218
**Physio Moderator**														
Motion (overall)	2.69	0.104	0.824	0.14	0.711	0.887	1.41	0.238	0.853	0.79	0.50	1.59	0.112	0.210
Breath Rate (first 2 min)	0.13	0.724	0.892	1.08	0.301	0.796	0.06	0.805	0.957	0.15	1.02	0.15	0.883	0.883
HR (first 1 min)	2.45	0.122	0.824	0.59	0.446	0.837	0.01	0.908	0.957	-0.70	0.29	-2.39	0.017	0.042
HRV (first 5 min)	0.03	0.853	0.892	4.04	0.048	0.362	0.00	0.957	0.957	42.30	65.70	0.64	0.520	0.650
Breath Rate change (last - first)	0.02	0.876	0.892	0.81	0.371	0.796	0.82	0.367	0.853	-0.19	0.82	-0.24	0.814	0.872
HR change (last - first)	0.17	0.686	0.892	2.44	0.123	0.508	0.72	0.398	0.853	-0.60	0.80	-0.75	0.455	0.620
HRV change (last - first)	0.68	0.412	0.892	2.28	0.135	0.508	0.55	0.461	0.865	9.92	38.69	0.26	0.798	0.872

Table 5H. Results from GLM tests of moderation. ‘DV’ refers to dependent variable of Awe from the post timepoint. ‘Psych Moderator’ and ‘Physio Moderator’ refer to either the psychological or physiological variable defined as the moderator. ‘Tech x Stim x Moderator’ refers to the model of a three-way interaction between the moderator and the group factors of Tech (MindGym vs VR) and Stimuli (Breathwork vs Rain). ‘Tech x Moderator’ refers to the model of a two-way interaction between the moderator and the group factor of Tech (MindGym vs VR). ‘Stim x Moderator’ refers to the model of a two-way interaction between the moderator and the group factor of Stimuli (Breathwork vs Rain). ‘Moderator’ refers to the effect of the moderator from the model of only main effects. ‘F’ refers to F-test from the GLM model. ‘b’ and ‘SE’ refer to the unstandardized beta coefficient and its standard error. ‘Z’ refers to the Z-test from the model. ‘p.raw’ and ‘p.fdr’ refer to the uncorrected p value and FDR-corrected p value.

Table 5I (EDI)

**DV**	**Tech x Stim x Moderator**			**Tech x Moderator**			**Stim x Moderator**			**Moderator**				
EDI	**F**	**p.raw**	**p.fdr**	**F**	**p.raw**	**p.fdr**	**F**	**p.raw**	**p.fdr**	**b**	**SE**	**Z**	**p.raw**	**p.fdr**
**Psych Moderator**														
DPES	0.25	0.617	0.891	0.55	0.461	0.894	1.09	0.299	0.499	0.54	0.27	2.03	0.043	0.128
Immersion	0.63	0.431	0.891	0.00	0.996	0.996	2.95	0.089	0.308	0.07	0.02	4.55	< .0001	< .001
IPQ (General)	0.00	0.949	0.949	4.05	0.047	0.351	5.88	0.017	0.257	0.32	0.13	2.49	0.013	0.048
IPQ (Involvement)	0.05	0.831	0.891	0.21	0.646	0.969	0.08	0.784	0.904	0.18	0.05	3.24	0.001	0.009
IPQ (Spatial Presence)	2.98	0.088	0.459	2.21	0.140	0.526	0.82	0.367	0.551	0.09	0.06	1.39	0.165	0.221
IPQ (Experienced Realism)	2.90	0.092	0.459	0.41	0.521	0.894	2.71	0.103	0.308	0.09	0.06	1.45	0.147	0.221
MODTAS	0.06	0.806	0.891	0.01	0.941	0.996	0.00	0.971	0.971	0.03	0.01	2.98	0.003	0.015
Openness	0.05	0.823	0.891	0.02	0.886	0.996	3.54	0.063	0.308	0.02	0.06	0.29	0.772	0.828
**Physio Moderator**														
Motion (overall)	2.08	0.153	0.573	0.03	0.862	0.996	1.76	0.188	0.402	-0.07	0.04	-1.83	0.067	0.168
Breath Rate (first 2 min)	4.48	0.038	0.459	5.40	0.023	0.345	1.93	0.170	0.402	0.10	0.07	1.35	0.177	0.221
HR (first 1 min)	1.25	0.269	0.673	0.79	0.379	0.894	1.16	0.286	0.499	-0.04	0.02	-1.60	0.110	0.203
HRV (first 5 min)	0.05	0.821	0.891	0.40	0.528	0.894	0.54	0.465	0.586	7.86	4.96	1.59	0.113	0.203
Breath Rate change (last - first)	1.58	0.213	0.639	0.11	0.745	0.996	0.53	0.469	0.586	-0.09	0.06	-1.55	0.122	0.203
HR change (last - first)	0.13	0.717	0.891	2.51	0.119	0.526	4.04	0.049	0.308	-0.05	0.06	-0.78	0.434	0.501
HRV change (last - first)	0.46	0.502	0.891	0.39	0.536	0.894	0.01	0.931	0.971	-0.54	2.88	-0.19	0.851	0.851

Table 5I. Results from GLM tests of moderation. ‘DV’ refers to dependent variable of EDI from the post timepoint. ‘Psych Moderator’ and ‘Physio Moderator’ refer to either the psychological or physiological variable defined as the moderator. ‘Tech x Stim x Moderator’ refers to the model of a three-way interaction between the moderator and the group factors of Tech (MindGym vs VR) and Stimuli (Breathwork vs Rain). ‘Tech x Moderator’ refers to the model of a two-way interaction between the moderator and the group factor of Tech (MindGym vs VR). ‘Stim x Moderator’ refers to the model of a two-way interaction between the moderator and

the group factor of Stimuli (Breathwork vs Rain). 'Moderator' refers to the effect of the moderator from the model of only main effects. 'F' refers to F-test from the GLM model. 'b' and 'SE' refer to the unstandardized beta coefficient and its standard error. 'Z' refers to the Z-test from the model. 'p.raw' and 'p.fdr' refer to the uncorrected p value and FDR-corrected p value.

Table 5J (BSE)

DV	Tech x Stim x Moderator			Tech x Moderator			Stim x Moderator			Moderator				
BSE	**F**	**p.raw**	**p.fdr**	**F**	**p.raw**	**p.fdr**	**F**	**p.raw**	**p.fdr**	**b**	**SE**	**Z**	**p.raw**	**p.fdr**
**Psych Moderator**														
DPES	0.81	0.371	0.888	0.57	0.452	0.974	2.60	0.110	0.671	-0.15	0.18	-0.83	0.409	0.767
Immersion	0.11	0.743	0.888	0.01	0.944	0.974	0.24	0.626	0.892	-0.02	0.01	-1.54	0.125	0.700
IPQ (General)	0.77	0.381	0.888	0.26	0.610	0.974	1.62	0.206	0.671	-0.16	0.09	-1.77	0.076	0.700
IPQ (Involvement)	0.86	0.355	0.888	5.47	0.021	0.315	2.30	0.132	0.671	0.03	0.04	0.84	0.404	0.767
IPQ (Spatial Presence)	0.09	0.761	0.888	0.69	0.409	0.974	0.40	0.528	0.888	0.01	0.05	0.16	0.876	0.898
IPQ (Experienced Realism)	1.26	0.263	0.888	0.80	0.374	0.974	2.07	0.153	0.671	0.04	0.04	0.97	0.330	0.767
MODTAS	0.01	0.912	0.965	1.32	0.253	0.974	0.96	0.329	0.705	0.00	0.01	-0.13	0.898	0.898
Openness	0.09	0.770	0.888	0.02	0.901	0.974	0.06	0.815	0.892	-0.01	0.04	-0.31	0.760	0.898
**Physio Moderator**														
Motion (overall)	0.09	0.766	0.888	0.56	0.457	0.974	0.03	0.862	0.892	-0.01	0.02	-0.29	0.769	0.898
Breath Rate (first 2 min)	0.18	0.677	0.888	0.03	0.862	0.974	0.12	0.726	0.892	1.00	79.00	1.26	0.265	0.767
HR (first 1 min)	0.00	0.965	0.965	1.80	0.184	0.974	1.25	0.268	0.671	0.00	0.02	0.15	0.884	0.898
HRV (first 5 min)	0.16	0.689	0.888	0.15	0.699	0.974	0.02	0.892	0.892	1.52	3.58	0.43	0.670	0.898
Breath Rate change (last - first)	0.52	0.473	0.888	0.02	0.895	0.974	1.37	0.246	0.671	0.06	0.04	1.48	0.140	0.700
HR change (last - first)	3.63	0.061	0.888	0.05	0.833	0.974	0.39	0.533	0.888	-0.04	0.04	-0.96	0.339	0.767
HRV change (last - first)	1.35	0.249	0.888	0.00	0.974	0.974	0.03	0.867	0.892	-0.33	2.10	-0.16	0.877	0.898

Table 5J. Results from GLM tests of moderation. 'DV' refers to dependent variable of BSE from the post timepoint. 'Psych Moderator' and 'Physio Moderator' refer to either the psychological or physiological variable defined as the moderator. 'Tech x Stim x Moderator' refers to the model of a three-way interaction between the moderator and the group factors of Tech (MindGym vs VR) and Stimuli (Breathwork vs Rain). 'Tech x Moderator' refers to the model of a two-way interaction between the moderator and the group factor of Tech (MindGym vs VR). 'Stim x Moderator' refers to the model of a two-way interaction between the moderator and the group factor of Stimuli (Breathwork vs Rain). 'Moderator' refers to the effect of the moderator from the model of only main effects. 'F' refers to F-test from the GLM model. 'b' and 'SE' refer to the unstandardized beta coefficient and its standard error. 'Z' refers to the Z-test from the model. 'p.raw' and 'p.fdr' refer to the uncorrected p value and FDR-corrected p value.
