# Supplementary material for: Contrasting cognitive, behavioral, and physiological responses to breathwork vs. naturalistic stimuli in reflective chamber and VR headset environments
Source: PLOS Ment Health. 2025 Mar 12;2(3):e0000269. doi: 10.1371/journal.pmen.0000269 (PMC12798627; doi:10.1371/journal.pmen.0000269)
Supplement: S6 Table — A. Descriptive Statistics. B. Repeated measures ANOVA. C. Post hoc comparisons. (PDF) [file pmen.0000269.s006.pdf]

S6: Follow-up Data

Table 6A (Descriptive Statistics)

		STAI		POMS		GA-VAS	
**Group**	**Timepoint**	**M**	**SD**	**M**	**SD**	**M**	**SD**
**MindGym (Breathwork)**	**Pre**	34.96	8.43	9.59	23.33	n/a	n/a
	**Post**	30.18	7.74	-0.86	18.51	n/a	n/a
	**FollowUp**	37.23	12.36	7.91	20.71	3.77	1.97
**MindGym (Rain)**	**Pre**	37.29	9.46	15.33	22.09	n/a	n/a
	**Post**	33.79	6.00	5.04	17.28	n/a	n/a
	**FollowUp**	39.63	11.61	10.54	17.39	4.25	2.19
**VR (Breathwork)**	**Pre**	34.10	7.44	7.05	19.11	n/a	n/a
	**Post**	29.10	4.67	-1.90	13.15	n/a	n/a
	**FollowUp**	36.30	7.61	4.65	12.53	3.95	1.85
**VR (Rain)**	**Pre**	33.24	9.04	6.38	27.12	n/a	n/a
	**Post**	32.95	6.52	2.10	22.07	n/a	n/a
	**FollowUp**	36.24	8.23	8.62	15.68	3.67	1.77

Table 6A. STAI = State Trait Anxiety Scale; POMS = Profile of Mood States (Total Mood Disturbance); GA-VAS = Generalized Anxiety - Visual Analog Scale; M = Mean SD = standard deviation

Table 6B (Repeated measures ANOVA)

	Timepoint						Timepoint x Tech					
**DV**	**df1**	**df2**	**F**	**p.raw**	**p.fdr**	**η^2_G**	**df1**	**df2**	**F**	**p.raw**	**p.fdr**	**η^2_G**
**STAI**	2.00	172.00	17.48	< .001	0.004	0.073	2.00	170.00	0.33	0.722	0.772	0.001
**POMS**	2.00	172.00	12.55	< .001	0.004	0.035	2.00	170.00	0.65	0.525	0.759	0.002
	**Timepoint x Stim**						**Timepoint x Tech x Stim**					
**DV**	**df1**	**df2**	**F**	**p.raw**	**p.fdr**	**η^2_G**	**df1**	**df2**	**F**	**p.raw**	**p.fdr**	**η^2_G**
**STAI**	2.00	170.00	1.24	0.293	0.586	0.006	2.00	166.00	0.42	0.664	0.772	0.002
**POMS**	2.00	170.00	0.21	0.811	0.811	0.001	2.00	166.00	0.57	0.569	0.759	0.002

Table 6B DV = dependent variable; STAI = State Trait Anxiety Scale; POMS = Profile of Mood States (Total Mood Disturbance); Timepoint = main effect model (within-subjects: pre, post, follow up); Timepoint x Tech = interaction model (between-groups: MindGym vs VR); Timepoint x Stim = interaction model (between-groups: Breathwork vs Rain); Timepoint x Tech x Stim = three-way interaction model; F = F-test with df1, df2 (degrees of freedom); P.RAW/p.fdr = uncorrected/FDR-corrected p-values

Table 6C (Post hoc comparisons)

	Post hoc tests by Timepoint (STAI)					
**Timepoints**	**ΔM**	**SE**	**df**	**t**	**Cohen's d**	**p.fdr**
**Post - Pre**	-3.39	0.99	172.00	-3.42	-0.40	0.002
**FollowUp - Pre**	2.45	0.99	172.00	2.47	0.29	0.015
**FollowUp - Post**	5.84	0.99	172.00	5.89	0.68	< .001
	**Post hoc tests by Timepoint (POMS)**					
**Timepoints**	**ΔM**	**SE**	**df**	**t**	**Cohen's d**	**p.fdr**
**Post - Pre**	-8.58	1.81	172.00	-4.74	-0.44	< .001
**FollowUp - Pre**	-1.76	1.81	172.00	-0.97	-0.09	0.332
**FollowUp - Post**	6.82	1.81	172.00	3.77	0.35	< .001

Table 6C STAI = State Trait Anxiety Scale; POMS = Profile of Mood States (Total Mood Disturbance); ΔM/SE = mean difference/standard error between timepoints; t, df = t-test statistic and degrees of freedom; Cohen's d = effect size; p.fdr = FDR-corrected p-value
