# Supplementary figures and images for: Contrasting cognitive, behavioral, and physiological responses to breathwork vs. naturalistic stimuli in reflective chamber and VR headset environments
Source: PLOS Ment Health. 2025 Mar 12;2(3):e0000269. doi: 10.1371/journal.pmen.0000269 (PMC12798627; doi:10.1371/journal.pmen.0000269)

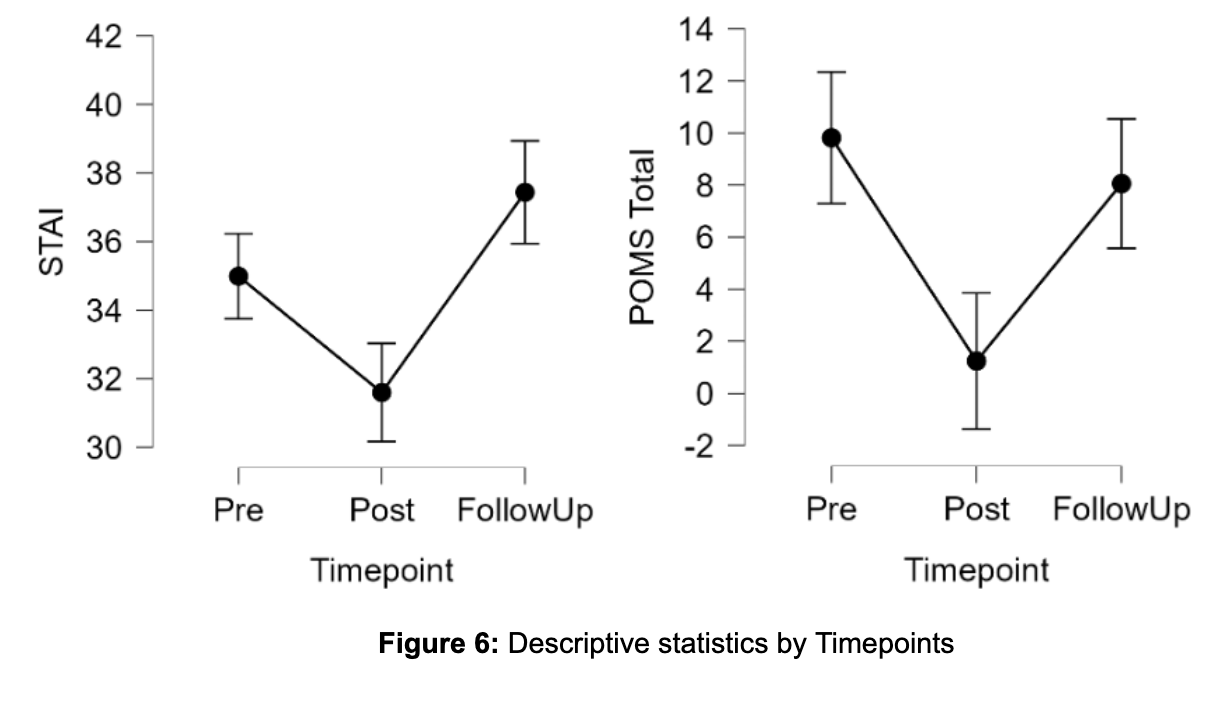

Supplement: S6 Fig — (TIFF) [file pmen.0000269.s007.tiff]
