# Supplementary material for: Contrasting cognitive, behavioral, and physiological responses to breathwork vs. naturalistic stimuli in reflective chamber and VR headset environments
Source: PLOS Ment Health. 2025 Mar 12;2(3):e0000269. doi: 10.1371/journal.pmen.0000269 (PMC12798627; doi:10.1371/journal.pmen.0000269)
Supplement: Attachment — Submitted filename: PLOS Simonian et al. - Response to Reviewers.docx [file pmen.0000269.s011.docx]

## **EDITOR**

**Thank you for submitting your manuscript to PLOS Mental Health. After careful consideration, we feel that it has merit but does not fully meet PLOS Mental Health’s publication criteria as it currently stands. Therefore, we invite you to submit a revised version of the manuscript that addresses the points raised during the review process.**

**The manuscript has been evaluated by two reviewers, and their comments are available below.**

**The reviewers have raised a number of major concerns. They request revisions to the data presentation and further consideration to potential limitations of the study, such as sample size and generalisability.**

**Could you please carefully revise the manuscript to address all comments raised?**

We are grateful for the thoughtful and constructive feedback from both reviewers. We have now added several complementary data presentations that enhance both immediate interpretability and deeper insights. These additions include: 1) a comprehensive repeated measures ANOVA table that provides at-a-glance understanding of our physiological outcomes, 2) detailed follow-up analyses with descriptive statistics and post-hoc comparisons, and 3) a thorough examination of participant demographics and cybersickness patterns that directly inform the real-world applicability of these technologies. To maintain manuscript clarity given these substantial additions, we have also condensed table captions throughout. We’ve also taken reviewer guidance and updated the limitations and future directions sections of the manuscript. Finally, we slightly updated the abstract to more closely reflect some of the points the reviewers brought up that actually serve as an angle of strength for the manuscript (that the anxiolytic effects originally generated from content in the MindGym were maintained via the 360 video recordings in VR). These enhancements make our findings more rapidly accessible to readers while providing critical new insights about implementation considerations, particularly regarding age and gender differences in technology tolerance. We appreciate this opportunity to reach PLOS Mental Health readers and remain open to any further edits or suggestions. Happy New Year!

## **Reviewer #1**

**Thank you for your submission. To enhance the clarity and comprehensiveness of your data presentation, I recommend adding the following tables:**

Thank you for your thoughtful review, which we agree has enhanced the clarity and comprehensiveness of the data presentation. Please see our responses and revisions below. **Repeated Measures ANOVA Table: Please include a table that presents the results of the repeated measures ANOVA. This table should detail the within-subjects and between-subjects effects, along with relevant F-values, degrees of freedom, p-values, and effect sizes.**

We have added a new Table 5 to the Results section under "Changes over time" which presents the comprehensive repeated measures ANOVA results for all physiological measures (HR, HRV, EDA, and BR). The analyses revealed significant main effects of time for heart rate (F(5.17,398.38) = 8.78, p < .001), electrodermal activity (F(2.35,162.37) = 17.11, p < .001), and breathing rate (F(2.02,171.33) = 25.31, p < .001), while heart rate variability showed no significant temporal changes. Notably, none of the physiological measures showed significant interactions with technology platform (VR vs. MindGym) or stimulus type (Breathwork vs. Rain), except for breathing rate which showed a significant interaction with stimulus type (F(2.48,208.49) = 28.49, p < .001), suggesting that the physiological effects were largely consistent across intervention conditions.

Table addition for manuscript:

**Table 5. Results from repeated measures ANOVA on physio measures.**

|  | **Timepoint** | | | | | | **Timepoint x Tech** | | | | | |
| --- | --- | --- | --- | --- | --- | --- | --- | --- | --- | --- | --- | --- |
| **DV** | **df1** | **df2** | **F** | **p.raw** | **p.fdr** | **η^2^_G_** | **df1** | **df2** | **F** | **p.raw** | **p.fdr** | **η^2^_G_** |
| **HR** | **5.17** | **398.38** | **8.78** | **< .001** | **0.004** | **0.005** | **5.14** | **390.59** | **0.56** | **0.738** | **0.777** | **0.003** |
| **HRV** | **1.00** | **77.00** | **1.54** | **0.219** | **0.438** | **0.009** | **1.00** | **76.00** | **0.31** | **0.581** | **0.615** | **0.002** |
| **EDA** | **2.35** | **162.37** | **17.11** | **< .001** | **0.004** | **0.020** | **2.35** | **159.51** | **0.92** | **0.414** | **0.552** | **0.001** |
| **BR** | **2.02** | **171.33** | **25.31** | **< .001** | **0.002** | **0.130** | **2.02** | **169.50** | **0.38** | **0.685** | **0.867** | **0.002** |
|  | **Timepoint x Stim** | | | | | | **Timepoint x Tech x Stim** | | | | | |
| **DV** | **df1** | **df2** | **F** | **p.raw** | **p.fdr** | **η^2^_G_** | **df1** | **df2** | **F** | **p.raw** | **p.fdr** | **η^2^_G_** |
| **HR** | **5.16** | **392.00** | **0.84** | **0.527** | **0.777** | **0.005** | **5.09** | **376.86** | **0.50** | **0.777** | **0.777** | **0.003** |
| **HRV** | **1.00** | **76.00** | **2.67** | **0.106** | **0.424** | **0.015** | **1.00** | **74.00** | **0.26** | **0.615** | **0.615** | **0.002** |
| **EDA** | **2.37** | **161.14** | **1.90** | **0.146** | **0.292** | **0.003** | **2.36** | **155.43** | **0.18** | **0.871** | **0.871** | **0.002** |
| **BR** | **2.48** | **208.49** | **28.49** | **< .001** | **0.002** | **0.140** | **2.48** | **203.01** | **0.19** | **0.867** | **0.867** | **0.001** |

DV: dependent variable; HR = heart rate; HRV = heart rate variability; EDA = electrodermal activity (tonic); BR = breath rate. Timepoint = main effect model (within-subjects); Timepoint x Tech = interaction model (between-groups: MindGym vs VR); Timepoint x Stim = interaction model (between-groups: Breathwork vs Rain); Timepoint x Tech x Stim = three-way interaction model. F = F-test with df1, df2 (degrees of freedom); P.RAW/P.FDR = uncorrected/FDR-corrected p-values. **Follow-Up Data Table: A table displaying follow-up data would help readers assess the sustainability of the intervention's effects or any time-related changes. Please include key descriptive and inferential statistics for each follow-up period.**

Thank you for this suggestion. We have added comprehensive follow-up data analyses in the Supplemental Materials: Tables 1-3; Figure 1, including descriptive statistics (Table 1), repeated measures ANOVA results (Table2), and post-hoc comparisons (Table 3). The data reveal significant temporal effects for both STAI (F(2,172) = 17.48, p < .001) and POMS (F(2,172) = 12.55, p < .001). While we observed immediate post-intervention improvements (consistent with the nonparametric timepoint effects we reported earlier in the manuscript), these effects were not sustained at one-week follow-up, with anxiety levels (STAI) actually exceeding baseline measurements and mood disturbance (POMS) returning to near-baseline levels. However, without also having measurements at one week *prior* to the start of the intervention (which would mirror the one week *post* intervention), it’s uncertain how these follow-up levels compare to subjects’ average mood states in general, irrespective of their participation in the experiment. This could be an interesting question for future studies.

We now include this text in the discussion portion of the manuscript:

Our follow-up analyses revealed that while the interventions produced significant immediate reductions in anxiety and mood disturbance, these beneficial effects were not maintained one week post-intervention. Particularly noteworthy was the observation that anxiety levels not only returned to baseline but exceeded pre-intervention measurements at follow-up, suggesting a potential rebound effect that warrants further investigation. These findings indicate that while single-session interventions can provide acute anxiety relief, maintaining long-term benefits may require regular practice or multiple sessions, especially given the apparent vulnerability to anxiety rebound effects. However, it's important to note that follow-up measurements were taken outside the controlled laboratory environment where pre- and post-intervention assessments occurred, potentially introducing different contextual factors and stressors. Furthermore, without also having measurements at one week *prior* to the start of the intervention (which would mirror the one week *post* intervention), it's uncertain how these follow-up levels compare to subjects' average mood states in general, irrespective of their participation in the experiment. This could be an interesting question for future studies.

We also included the following additional tables and figures in the supplemental information and refer readers there in the main text:

**Supplemental Materials: Follow-up Data**

**Supplemental Table 6A (Descriptive Statistics)**

|  |  | **STAI** | | **POMS** | | **GA-VAS** | |
| --- | --- | --- | --- | --- | --- | --- | --- |
| **Group** | **Timepoint** | **M** | **SD** | **M** | **SD** | **M** | **SD** |
| **MindGym (Breathwork)** | **Pre** | 34.96 | 8.43 | 9.59 | 23.33 | n/a | n/a |
|  | **Post** | 30.18 | 7.74 | -0.86 | 18.51 | n/a | n/a |
|  | **FollowUp** | 37.23 | 12.36 | 7.91 | 20.71 | 3.77 | 1.97 |
| **MindGym (Rain)** | **Pre** | 37.29 | 9.46 | 15.33 | 22.09 | n/a | n/a |
|  | **Post** | 33.79 | 6.00 | 5.04 | 17.28 | n/a | n/a |
|  | **FollowUp** | 39.63 | 11.61 | 10.54 | 17.39 | 4.25 | 2.19 |
| **VR (Breathwork)** | **Pre** | 34.10 | 7.44 | 7.05 | 19.11 | n/a | n/a |
|  | **Post** | 29.10 | 4.67 | -1.90 | 13.15 | n/a | n/a |
|  | **FollowUp** | 36.30 | 7.61 | 4.65 | 12.53 | 3.95 | 1.85 |
| **VR (Rain)** | **Pre** | 33.24 | 9.04 | 6.38 | 27.12 | n/a | n/a |
|  | **Post** | 32.95 | 6.52 | 2.10 | 22.07 | n/a | n/a |
|  | **FollowUp** | 36.24 | 8.23 | 8.62 | 15.68 | 3.67 | 1.77 |

**Supplemental Table 6A.** STAI = State Trait Anxiety Scale; POMS = Profile of Mood States (Total Mood Disturbance); GA-VAS = Generalized Anxiety - Visual Analog Scale; M = mean; SD = standard deviation

**Supplemental Figure 6A (Descriptive statistics by Timepoints)**

**Supplemental Table 6B (Repeated measures ANOVA)**

|  | **Timepoint** | | | | | | **Timepoint x Tech** | | | | | |
| --- | --- | --- | --- | --- | --- | --- | --- | --- | --- | --- | --- | --- |
| **DV** | **df1** | **df2** | **F** | **p.raw** | **p.fdr** | **η^2^_G_** | **df1** | **df2** | **F** | **p.raw** | **p.fdr** | **η^2^_G_** |
| **STAI** | 2.00 | 172.00 | 17.48 | < .001 | 0.004 | 0.073 | 2.00 | 170.00 | 0.33 | 0.722 | 0.772 | 0.001 |
| **POMS** | 2.00 | 172.00 | 12.55 | < .001 | 0.004 | 0.035 | 2.00 | 170.00 | 0.65 | 0.525 | 0.759 | 0.002 |
|  | **Timepoint x Stim** | | | | | | **Timepoint x Tech x Stim** | | | | | |
| **DV** | **df1** | **df2** | **F** | **p.raw** | **p.fdr** | **η^2^_G_** | **df1** | **df2** | **F** | **p.raw** | **p.fdr** | **η^2^_G_** |
| **STAI** | 2.00 | 170.00 | 1.24 | 0.293 | 0.586 | 0.006 | 2.00 | 166.00 | 0.42 | 0.664 | 0.772 | 0.002 |
| **POMS** | 2.00 | 170.00 | 0.21 | 0.811 | 0.811 | 0.001 | 2.00 | 166.00 | 0.57 | 0.569 | 0.759 | 0.002 |

**Supplemental Table 6B.** DV = dependent variable; STAI = State Trait Anxiety Scale; POMS = Profile of Mood States (Total Mood Disturbance); Timepoint = main effect model (within-subjects: pre, post, follow up); Timepoint x Tech = interaction model (between-groups: MindGym vs VR); Timepoint x Stim = interaction model (between-groups: Breathwork vs Rain); Timepoint x Tech x Stim = three-way interaction model; F = F-test with df1, df2 (degrees of freedom); P.RAW/p.fdr = uncorrected/FDR-corrected p-values

**Supplemental Table 6C (Post hoc comparisons)**

|  | **Post hoc tests by Timepoint (STAI)** | | | | | |
| --- | --- | --- | --- | --- | --- | --- |
| **Timepoints** | **ΔM** | **SE** | **df** | **t** | **Cohen's d** | **p.fdr** |
| **Post - Pre** | -3.39 | 0.99 | 172.00 | -3.42 | -0.40 | 0.002 |
| **FollowUp - Pre** | 2.45 | 0.99 | 172.00 | 2.47 | 0.29 | 0.015 |
| **FollowUp - Post** | 5.84 | 0.99 | 172.00 | 5.89 | 0.68 | < .001 |
|  | **Post hoc tests by Timepoint (POMS)** | | | | | |
| **Timepoints** | **ΔM** | **SE** | **df** | **t** | **Cohen's d** | **p.fdr** |
| **Post - Pre** | -8.58 | 1.81 | 172.00 | -4.74 | -0.44 | < .001 |
| **FollowUp - Pre** | -1.76 | 1.81 | 172.00 | -0.97 | -0.09 | 0.332 |
| **FollowUp - Post** | 6.82 | 1.81 | 172.00 | 3.77 | 0.35 | < .001 |

**Supplemental Table 6C.** STAI = State Trait Anxiety Scale; POMS = Profile of Mood States (Total Mood Disturbance); ΔM/SE = mean difference/standard error between timepoints; t, df = t-test statistic and degrees of freedom; Cohen's d = effect size; p.fdr = FDR-corrected p-value

**Dropout Data Table: Providing a table that outlines dropout rates and reasons for attrition will add transparency to the study's methodology. Including demographics of those who dropped out versus those who completed the study could also be helpful.**

Thank you for your thoughtful suggestion regarding dropout rates and demographic information.

We now include the following information in the manuscript:

“Of the 126 enrolled participants, 87 completed the one-week survey (See Table 3). The similar distribution of demographic characteristics suggests random attrition, though this conclusion is limited by incomplete demographic data across the full sample.”

**Table 3. Study participants by follow-up completion status across groups and demographics**

|  |  | **Completers (N=49)** | **Non-completers (N=24)** |
| --- | --- | --- | --- |
| **Group** |  |  |  |
|  | **VR (Breathwork)** | 12 | 9 |
|  | **VR (Rain)** | 9 | 6 |
|  | **MindGym (Breathwork)** | 13 | 5 |
|  | **MindGym (Rain)** | 15 | 4 |
| **Gender** |  |  |  |
|  | **Male** | 25 | 10 |
|  | **Female** | 24 | 14 |
| **Education** |  |  |  |
|  | **Bachelor’s degree** | 27 | 13 |
|  | **Master’s degree** | 9 | 3 |
|  | **Some college** | 6 | 5 |
|  | **High school diploma** | 5 | 1 |
|  | **GED or Equivalent** | 0 | 1 |
|  | **Professional degree** | 1 | 0 |
|  | **Associate’s degree** | 1 | 1 |
| **Race** |  |  |  |
|  | **White** | 23 | 11 |
|  | **Asian** | 10 | 9 |
|  | **Multiracial** | 6 | 3 |
|  | **Black/African American** | 2 | 1 |
|  | **Hispanic/Latino** | 4 | 0 |
|  | **Indigenous American** | 1 | 0 |
|  | **Not disclosed** | 3 | 0 |
| **Ethnicity** |  |  |  |
|  | **Not Hispanic/Latinx** | 39 | 23 |
|  | **Hispanic/Latinx** | 9 | 1 |
|  | **Unknown** | 1 | 0 |

Note: Due to a clerical error during data preparation, demographic data were available for only 73 of 126 enrolled participants (57.9 %). Of the total enrolled sample, 87 participants (69.0%) completed the one-week follow-up survey. Similar distributions of demographic characteristics between completers and non-completers suggest random attrition, though this conclusion is limited by incomplete demographic data across the full sample. The mean age for completers were 43.63 years and 35.58 years for non-completers.

The inclusion of this additional data prompted the inclusion of additional commentary in the discussion section:

The most notable difference was age, with survey completers being about 8 years older on average (43.6 vs 35.6 years), while other demographic differences were relatively modest in comparison. Another group difference was also observed in intervention type, with MindGym conditions showing notably higher completion rates (76% combined) compared to VR conditions (58% combined).

**These additions will strengthen the presentation of your findings and offer readers a more detailed understanding of your results.**

We appreciate the reviewer's constructive suggestion to enhance the presentation of our findings. These additions improve both the immediate interpretability of our results and facilitate future meta-analyses by providing clear, accessible data points throughout the manuscript.

## **Reviewer #2**

**In my perspective, the study addresses an innovative and timely area in mental health research, exploring the comparative efficacy of VR and physical immersive environments for including anxiety reduction, mood enhancement, and cognitive function improvement. Combining VR and a physical reflective chamber with two distinct stimuli (rain and breathwork) and examining their effect on cognitive and physiological outcomes adds to the novelty. Particularly, the use of individual differences like MODTAS and Openness to explain responses is a novel approach to personalize treatment, which may be of significant interest in precision mental health interventions.**

We are grateful for the reviewer's astute recognition of our study's core aim: to advance personalized approaches to mental wellness through understanding individual differences in response to immersive experiences. Their insights regarding the potential implications for precision mental health interventions directly align with our institute's mission to develop evidence-based, individually-tailored pathways to well-being. We have incorporated their thoughtful suggestions throughout the manuscript to better articulate these important implications. Your comments around the 360 video as a limitation also prompted an additional reworking of some text in the manuscript to also showcase this as a strength by focusing on the fact that the experience was originally created in the MindGym and then ported to VR and that the anxiolytic effects were preserved.
 **The methodology appears robust, using a randomized assignment of 126 participants to four conditions, which allows for control over confounding variables and increases confidence in the study's findings. The use of both physiological and psychological measures (cognitive tests, breath rate, and anxiety scales) offers a well-rounded assessment of the interventions' impact. However, it might be worth noting a few points for potential improvement, for example sample size and generalizability: although 126 participants is a decent sample size, understanding if the sample is representative (e.g., age, gender, socioeconomic status, previous experience with VR, mental health status…) would help assess the generalizability of the findings. Moreover, assessing the presence of cybersickness or satisfaction with the digital environments could provide valuable insights, as these factors may have influenced participants' responses and overall effectiveness of the interventions. These points could also be a limitation and not only the use of a 360º video instead of a VR condition to compare to MindGym.**

Thank you for your thoughtful comments regarding sample representativeness. We agree that understanding the demographic composition of our sample is important for assessing generalizability. While we encountered a clerical error during data preparation that resulted in incomplete demographic data collection, we can provide information for the 74 participants (58.7% of our sample) for whom we have demographic data.

Among these participants, education levels varied from high school to advanced degrees: 43 held Bachelor's degrees, 15 had Master's degrees, 8 had some college but no degree, 6 had high school diplomas, 1 had an Associate's degree, and 1 had a professional degree. The racial composition included 36 White participants, 20 Asian, 10 multiracial, 3 Black or African American, 2 Hispanic or Latino, 1 Indigenous American, and 2 who preferred not to answer. Regarding ethnicity, 63 identified as Not Hispanic or Latinx, 10 as Hispanic or Latinx, and 1 as unknown. We included this information into the methods (participants) section of the manuscript.

From our complete dataset (n=126), we can report age (range: 18-74 years, mean = 42.3 years) and gender distribution (54% female, 46% male). While we acknowledge that our incomplete demographic data collection limits our ability to fully assess sample representativeness, the available data suggests our sample included participants from diverse educational, racial, and ethnic backgrounds. We recognize this limitation and have noted it in our discussion section.

In response to the reviewer's concerns about cybersickness and its potential influence on intervention effectiveness, we conducted a comprehensive analysis of motion sickness, including sub components of gastric, central, peripheral, and sopite symptoms across different age groups (18-34, 35-54, 55+) and genders. Our findings revealed distinct patterns that have important implications for both the interpretation of our current results and future virtual environment interventions. We included this information into the findings and discussion section of the manuscript:

"Contrary to our expectations, young adults (18-34) showed higher motion sickness susceptibility (mean = 20.58, SD = 13.74) than middle-aged (mean = 15.73, SD = 4.80) and older adults (mean = 15.02, SD = 3.55). Additionally, MindGym conditions unexpectedly showed slightly higher motion sickness scores (Breathwork: mean = 19.00, Rain: mean = 17.80) compared to VR conditions (Breathwork: mean = 16.81, Rain: mean = 16.83). While this appears to contradict established theories about vestibular mismatch in VR environments (Reggente et al., 2018), it's important to note that our use of 360° video rather than fully interactive VR may have created a different type of perceptual experience than typically studied in VR research, as the non-interactive nature of both conditions could have affected motion sickness in ways not predicted by traditional vestibular mismatch theory. Males and females showed similar total motion sickness scores (males: mean = 17.36, females: mean = 17.85), though females exhibited greater variability across symptom categories. Sopite symptoms remained relatively consistent across age groups (mean ≈ 23.0), with both conditions showing similar levels (MindGym: mean = 22.66, VR: mean = 23.41), with a slight advantage to the MindGym, making it potentially more ideal for reliably inducing mood states.

The unexpected age and platform differences in motion sickness susceptibility challenge conventional assumptions about cybersickness in younger populations and digital environments. The substantial individual variability in symptoms, particularly among younger adults and across platforms, represents a study limitation and emphasizes the need for personalized comfort settings and adaptive monitoring systems in future research designs. Additionally, future studies should consider implementing both fully interactive VR environments and more detailed measures of cybersickness and environmental satisfaction to better understand how these factors might influence intervention effectiveness. Such modifications would help distinguish intervention effectiveness from cybersickness effects while accounting for unexpected susceptibility patterns across different types of digital experiences."

The following table is added to the “Descriptives” section of the Findings.

**Table 7. Motion sickness scores across demographics and groups**

| **Motion Sickness** | | **Total Score** | | **Gastrointestinal** | | **Central** | | **Peripheral** | | **Sopite-related** | |
| --- | --- | --- | --- | --- | --- | --- | --- | --- | --- | --- | --- |
|  |  | **Mean** | **SD** | **Mean** | **SD** | **Mean** | **SD** | **Mean** | **SD** | **Mean** | **SD** |
| **Age** |  |  |  |  |  |  |  |  |  |  |  |
|  | **18-34** | 20.58 | 13.74 | 17.19 | 14.63 | 20.38 | 15.03 | 18.24 | 16.43 | 26.00 | 17.87 |
|  | **35-54** | 15.73 | 4.80 | 12.47 | 4.63 | 15.45 | 5.96 | 14.13 | 7.15 | 20.54 | 9.93 |
|  | **55+** | 15.02 | 3.55 | 11.57 | 2.55 | 14.67 | 5.27 | 11.73 | 1.71 | 21.39 | 9.57 |
| **Gender** |  |  |  |  |  |  |  |  |  |  |  |
|  | **Female** | 17.85 | 9.36 | 13.84 | 8.52 | 17.35 | 10.22 | 15.46 | 12.33 | 24.25 | 14.78 |
|  | **Male** | 17.36 | 10.26 | 14.64 | 11.71 | 17.32 | 11.65 | 15.11 | 11.18 | 21.83 | 13.01 |
| **Group** |  |  |  |  |  |  |  |  |  |  |  |
|  | **Cube** | 18.39 | 10.37 | 15.43 | 11.84 | 18.52 | 12.42 | 16.40 | 13.63 | 22.66 | 11.47 |
|  | **VR** | 16.82 | 9.17 | 13.05 | 8.19 | 16.16 | 9.11 | 14.17 | 9.42 | 23.41 | 16.09 |
|  | **Breath** | 17.87 | 10.62 | 14.45 | 9.94 | 17.71 | 12.23 | 16.72 | 12.98 | 22.35 | 14.03 |
|  | **Rain** | 17.33 | 8.92 | 14.02 | 10.56 | 16.95 | 9.46 | 13.80 | 10.15 | 23.75 | 13.88 |
|  | **MindGym (Breathwork)** | 19.00 | 10.18 | 15.23 | 9.13 | 19.50 | 13.95 | 17.32 | 13.57 | 23.39 | 12.08 |
|  | **MindGym (Rain)** | 17.80 | 10.69 | 15.63 | 14.12 | 17.57 | 10.89 | 15.51 | 13.84 | 21.96 | 10.98 |
|  | **VR (Breathwork)** | 16.81 | 11.08 | 13.72 | 10.73 | 16.03 | 10.30 | 16.16 | 12.59 | 21.38 | 15.77 |
|  | **VR (Rain)** | 16.83 | 6.68 | 12.31 | 3.91 | 16.30 | 7.77 | 11.98 | 2.32 | 25.65 | 16.40 |

Age groups are divided into 18-34, 35-54, and 55+ years.. M and SD represent the mean and standard deviation **It would be interesting to see the study discuss practical implications for mental health professionals, particularly those working with clients with anxiety, as this could be highly relevant to readers of PLOS Mental Health.**

Thank you for this insightful suggestion. We agree that discussing practical clinical applications would enhance the manuscript's relevance for mental health practitioners. We have added the following to our future directions section:

"Future research should explore the potential utility of brief digital relaxation interventions as pre-session tools in clinical settings. Given the potent, acute anxiolytic effects observed in our study, these interventions could help standardize patients' arousal states before therapy sessions, potentially offsetting situational stressors (e.g., commute-related anxiety) that might otherwise impact session effectiveness. Such application could be particularly valuable in urban clinical settings where external stressors are common. Investigating whether these interventions can create a more consistent baseline state for therapeutic engagement would be an important next step in evaluating their ecological utility in clinical practice." **Line 741: authors should not use contractions “weren’t seen”, please change to “were not see”.**

Thank you for the attention to detail. We’ve reviewed and updated the manuscript to remove all contractions.
